# Differences in Coformer Interactions of the 2,4-Diaminopyrimidines Pyrimethamine and Trimethoprim

**DOI:** 10.1021/acs.cgd.2c00035

**Published:** 2022-04-08

**Authors:** Lamis Alaa Eldin Refat, Ciaran O’Malley, John M. Simmie, Patrick McArdle, Andrea Erxleben

**Affiliations:** † School of Chemistry, National University of Ireland Galway, Galway H91TK33, Ireland; ‡ Synthesis and Solid State Pharmaceutical Centre (SSPC), Limerick V94 T9PX, Ireland

## Abstract

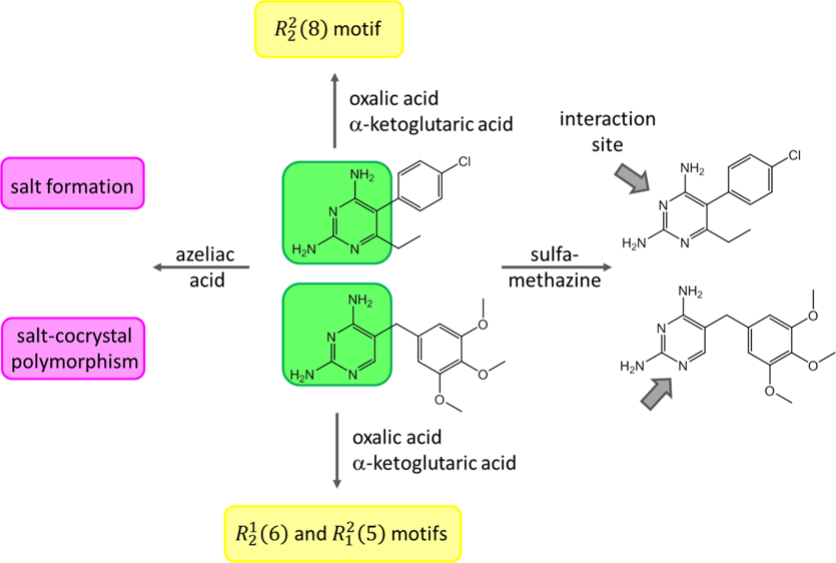

The identification and study of supramolecular synthons is a fundamental task in the design of pharmaceutical cocrystals. The malaria drug pyrimethamine (pyr) and the antibiotic trimethoprim (tmp) are both 2,4-diaminopyrimidine derivatives, providing the same C–NH_2_/N=C/C–NH_2_ and C–NH_2_/N=C interaction sites. In this article, we analyze and compare the synthons observed in the crystal structures of tmp and pyr cocrystals and molecular salts with sulfamethazine (smz), α-ketoglutaric acid (keto), oxalic acid (ox), sebacic acid (seb), and azeliac acid (az). We show that the same coformer interacts with different binding sites of the 2,4-diaminopyrimidine ring in the respective tmp and pyr cocrystals or binds at the same site but gives H bonding patterns with different graph set notions. Pyr·smz·CH_3_OH is the first crystal structure in which the interaction of the sulfa drug at the C–NH_2_/N=C/C–NH_2_ site with three parallel NH_2_···N, N···NH_sulfonamide_, and NH_2_···O=S H bonds is observed. The main synthon in (tmp^+^)(keto^–^).0.5H_2_O and (tmp^+^)_2_(ox^2–^)·2CH_3_OH is the motif of fused *R*_2_^1^(6) and *R*_1_^2^(5) rings instead of the *R*_2_^2^(8) motif typically observed in tmp^+^ and pyr^+^ carboxylates. Tmp/az is a rare example of cocrystal-salt polymorphism where the two solid-state forms have the same composition, stoichiometry, and main synthon. Theoretical calculations were performed to understand the order of stability, which is tmp·az cocrystal > (tmp^+^)(az^–^) salt. Finally, two three-component tmp/sulfa drug/carboxylate cocrystals with a unique ternary synthon are described.

## Abstract 2

Differences and similarities in coformer interactions with the 2,4-diaminopyrimidine entities in pyrimethamine and trimethoprim are discussed.

## Introduction

The development of cocrystals is largely based on crystal engineering principles. In crystal engineering, supramolecular synthons between functional groups are used to manipulate the intermolecular interactions. By controlling the crystal packing, cocrystals with the desired properties can be designed. The largest and most widely studied class of cocrystals are pharmaceutical cocrystals, that is, two drugs or one drug and a pharmaceutically acceptable coformer held together in the same crystal lattice by noncovalent interactions.^1−4^ Cocrystallization allows for the manipulation and optimization of the physicochemical properties of an active pharmaceutical ingredient (API) such as chemical and physical stability, hygroscopicity, solubility, and dissolution behavior without the need for chemically modifying the API molecule. APIs often have more than one functional group that can interact with coformers. Knowledge of the hierarchy of synthons is crucial to the rational design of new cocrystals. Furthermore, the presence of different functional groups or interaction sites can be exploited in the synthesis of higher-order cocrystals.^5−17^ Cocrystals containing more than two components are significantly more difficult to isolate than binary cocrystals because not only the supramolecular synthons but also the size, shape, and solubility of the three components must match and there is always the risk that a more stable binary cocrystal crystallizes instead.

**Figure 1:**
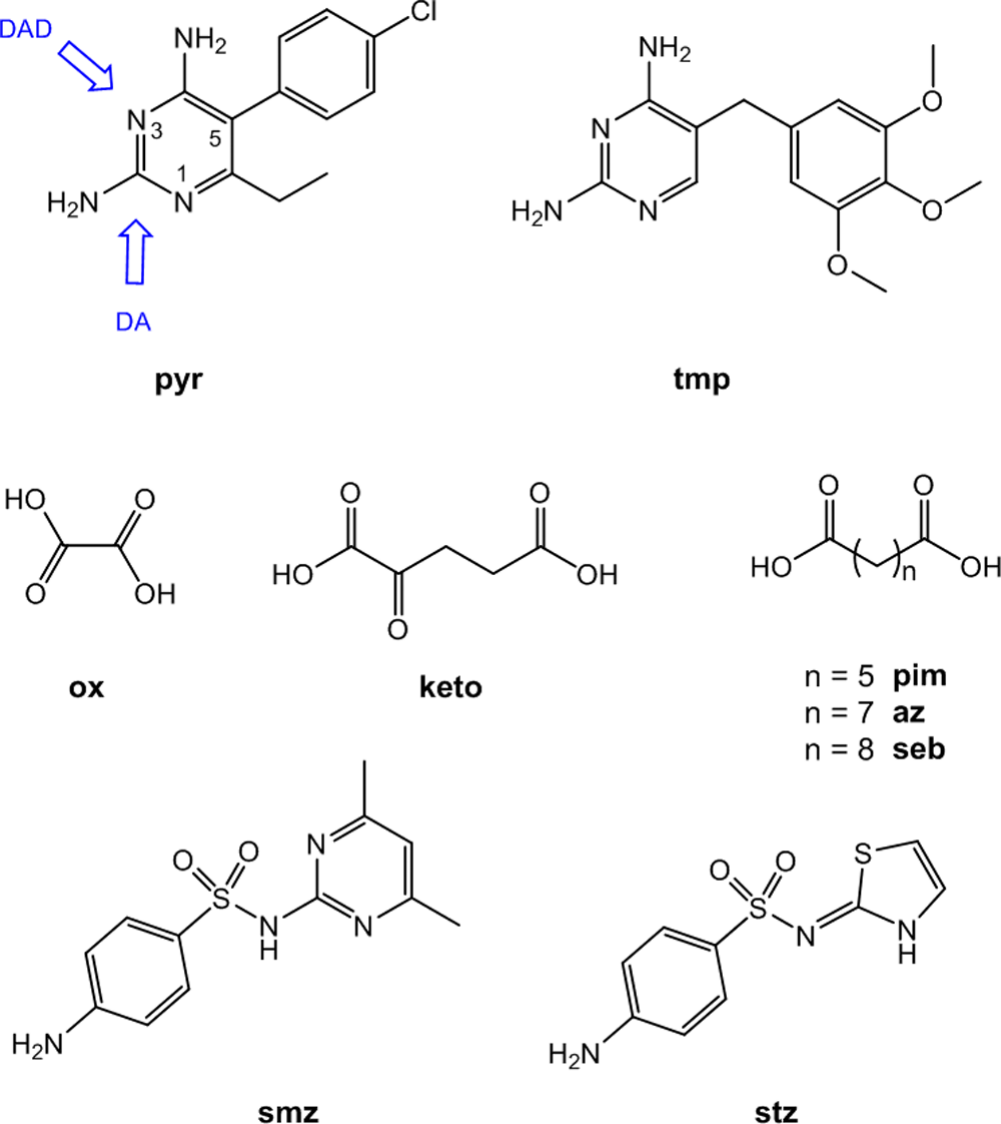
Chemical structures and H bond donor (D) and acceptor (A) sites of pyrimethamine, trimethoprim, and the coformers used in this study.

We have recently studied the formation of binary and ternary cocrystals of the malaria drug pyrimethamine (pyr, Figure 1).^18^ The 2,4-diaminopyrimidine ring of pyr provides two distinct interaction sites for coformers, the C2–NH_2_/N3/C4–NH_2_ (donor–acceptor–donor, DAD) site and the C2–NH_2_/N1 (donor–acceptor, DA) site. We have investigated combinations of various ADA and AD coformers and carried out competition experiments with different AD coformers such as saccharin and monocarboxylic acids. We have now included dicarboxylic acid coformers and obtained more binary and ternary cocrystals of pyr with new structural motifs. The identification of new synthons and the understanding of synthon preferences is a fundamental objective of crystal engineering. We also report a cocrystallization study of the related 2,4-diaminopyrimidine derivative trimethoprim (tmp, Figure 1). Tmp is an antibiotic mainly used for the treatment of urinary tract infections. A few cocrystals and salts of tmp have been reported previously.^19−26^ The crystal structure determination and the comparison of pyr- and tmp-coformer systems described in our present study revealed several interesting observations. (1) In contrast to pyr that formed a 1:1 salt with azeliac acid (az) as the stable form, the tmp/az system is one of the few examples of salt-cocrystal polymorphism. Polymorphism in multicomponent systems and the crystallization of solid-state forms with different ionization states are well established. However, systems with the same composition, stoichiometry, and the same main synthon that differ only in the location of the acidic proton at the same temperature are rare.^27−30^ (2) While the sulfa drug sulfamethazine (smz) interacts with the DA binding site of tmp—as observed for all previously reported tmp-sulfa drug cocrystals^19−23^—a new synthon between smz and the DAD site of the 2,4-diaminopyrimidine ring is found in pyr·smz, namely, three parallel NH_2_···N, N···NH_sulfonamide_, and NH_2_···O=S H bonds. Oxalic acid (ox) and α-ketoglutaric acid (keto) also form different H bonding motifs with the 2,4-diaminopyrimidine rings of tmp and pyr (fused *R*_2_^1^(6) and *R*_1_^2^(5) rings vs *R*_2_^2^(8) rings). (3) Contrary to our expectation based on (2) that the cocrystallization with both a carboxylic acid (AD coformer) and a sulfa drug (ADA coformer) would give a ternary cocrystal with the two coformers occupying the two 2,4-diaminopyrimidine binding sites, crystal structure determination of (tmp^+^)(pim^–^)·stz and (tmp^+^)(seb^–^)·stz·2H_2_O·C_3_H_6_O (pim = pimelic acid, seb = sebacic acid, stz = sulfathiazole) revealed a unique ternary synthon in which the S=O and imido NH of the sulfa drug H bond to the amino nitrogen and carboxylate oxygen of the tmp^+^···carboxylate pair. The reasons for the differences in the crystallization behavior of the closely related tmp and pyr molecules are discussed.

## Experimental Section

### Materials

pyr, tmp, keto, az, pim, and seb were purchased from Tokyo Chemical Industry, Europe. ox and smz were obtained from Sigma-Aldrich. Sulfathiazole (stz) was obtained from Fluka Analytical. The solvents acetonitrile, methanol (Merck Millipore), ethyl acetate (Sigma-Aldrich), and acetone (Fisher Chemical) were of analytical grade and were used as received.

### Solution Crystallization

25 mg of pyr or tmp and 1 mol equiv of the respective coformer(s) were dissolved in the minimum amount of solvent, and the solvent was allowed to slowly evaporate from an open vial. Crystallization experiments were carried out in methanol, acetonitrile, acetone, and ethyl acetate. Within a few days, X-ray suitable single crystals of (pyr^+^)_2_(ox^2–^)·1.5H_2_O, (tmp^+^)_2_(ox^2–^)·2CH_3_OH, (tmp^+^)_2_(az^2–^)·tmp·6H_2_O, tmp·pyr·H_2_O, pyr·smz·CH_3_OH, and (tmp^+^)_2_(seb^2–^)·2CH_3_OH·2H_2_O were obtained from methanol. (Tmp^+^)_2_(ox^2–^)·6.5H_2_O, (tmp^+^)(keto^–^)·0.5H_2_O, tmp.az, pyr.0.5seb·CH_3_CN, tmp·pim.0.5CH_3_CN, (pyr^+^)(az^–^) form I, and (tmp^+^)(pim^–^)·stz crystallized from acetonitrile, while (tmp^+^)(az^–^) and (tmp^+^)_2_(seb^2–^)·2stz·2H_2_O·C_3_H_6_O crystallized from ethyl acetate and acetone, respectively.

### Ball-Milling

Equimolar mixtures of tmp or pyr and the respective coformer(s) (120–150 mg in total, Table S1) were placed in 2 mL Eppendorf tubes. 20 μL of solvent and one 5 mm diameter stainless steel ball were added to each sample. The Eppendorf tubes were placed in an in-house 3D-printed six-tube sample holder. An oscillatory ball mill (Mixer Mill MM400, Retsch GmbH & Co., Germany) was used to mill the samples at 25 Hz for 20 min. The milled powder samples were analyzed immediately by X-ray powder diffraction.

### Crystallization by Sublimation

The salt (pyr^+^)(az^–^) form II was crystallized from the gas phase using an in-house sublimation apparatus.^31^ Equimolar ratios of pyr and az were sublimed from both ends of a standard 15 × 160 mm test tube sealed under vacuum. Two heaters were used to sublime the components, and the temperatures at the pyr and az ends were set at 193.5 and 148.3 °C, respectively. Pyr and az formed small crystals of (pyr^+^)(az^–^) form II in the middle of the test tube after 17 h.

### Slurry Experiments

Competitive slurry experiments were carried out for (tmp^+^)_2_(az^2–^)·tmp·6H_2_O and tmp·az to determine the most stable polymorph. 20 mg of each sample was mixed with 1 mL of methanol in a 10 mm diameter glass vial and the mixture was stirred for 48 h at room temperature, followed by drying in a vacuum oven at 40 °C for 6 h and recording the powder X-ray pattern.

### Differential Scanning Calorimetry

Differential scanning calorimetry (DSC) was carried out in open aluminum crucibles using an STA625 thermal analyzer (Rheometric Scientific, Piscataway, New Jersey). The DSC plots were recorded between 20 and 300 °C with a heating rate of 10 °C/min. Nitrogen was purged in ambient mode, and an indium standard was used for calibration.

### X-ray Powder Diffraction

X-ray powder patterns were recorded on an Inel Equinox 3000 powder diffractometer (Artenay, France). Cu K_α_ radiation (λ = 1.54178 Å, 35 kV, 25 mA) was used, and data were collected between 2θ 5 and 90°. Theoretical powder patterns were calculated from the single crystal X-ray data using the Oscail software package.^32^

### Single Crystal X-ray Analysis

Single crystal X-ray data were collected on an Oxford Diffraction Xcalibur system (Oxfordshire, UK). The crystal structures of tmp·pyr·H_2_O, (tmp^+^)(keto^–^)·0.5H_2_O, pyr·smz·CH_3_OH, (pyr^+^)_2_(ox^2–^)·1.5H_2_O, (tmp^+^)_2_(ox^2–^)·2CH_3_OH, (tmp^+^)_2_(ox^2–^)·6.5H_2_O, (pyr^+^)(az^–^) form I, (pyr^+^)(az^–^) form II, tmp·az, (tmp^+^)(az^–^), (tmp^+^)_2_(az^2–^)·tmp·6H_2_O, (tmp^+^)_2_(seb^2–^)·2CH_3_OH·2H_2_O, pyr.0.5seb·CH_3_CN, tmp·pim.0.5CH_3_CN, (tmp^+^)(pim^–^)·stz, and (tmp^+^)_2_(seb^2–^)·2stz·2H_2_O·C_3_H_6_O were solved by direct methods using SHELXT and refined using SHELXL 2018/3 within the Oscail package.^32−34^ Crystallographic data and details of refinement are listed in Tables S2–S4. The cif files can be obtained free of charge at www.ccdc.cam.ac.uk/conts/retrieving.html or from the Cambridge Crystallographic Data Centre, Cambridge, UK with the REF codes 2129639–2129654.

### Calculations

CASTEP version 20.11^35^ was used to calculate lattice enthalpies with fixed unit cell dimensions using a PC or the ICHEC National HPC service. Kpoints were calculated with a “minidistance” of 20 Å using getKPoints software.^36^ Input files for CASTEP were generated using Oscail software,^37^ and CASTEP was run on a PC using the windows subsystem for Linux. The PBEsol functional^38^ was used in combination with the Tkatchenko and Scheffler,^39^ and dispersion correction and on-the-fly norm conserving pseudopotentials were employed. A plane-wave basis-set cutoff of 843 eV was used in all calculations. The energy per formula unit was given by *E*_cell_/*Z*.

## Results and Discussion

### Tmp·pyr Cocrystal

**Figure 2:**
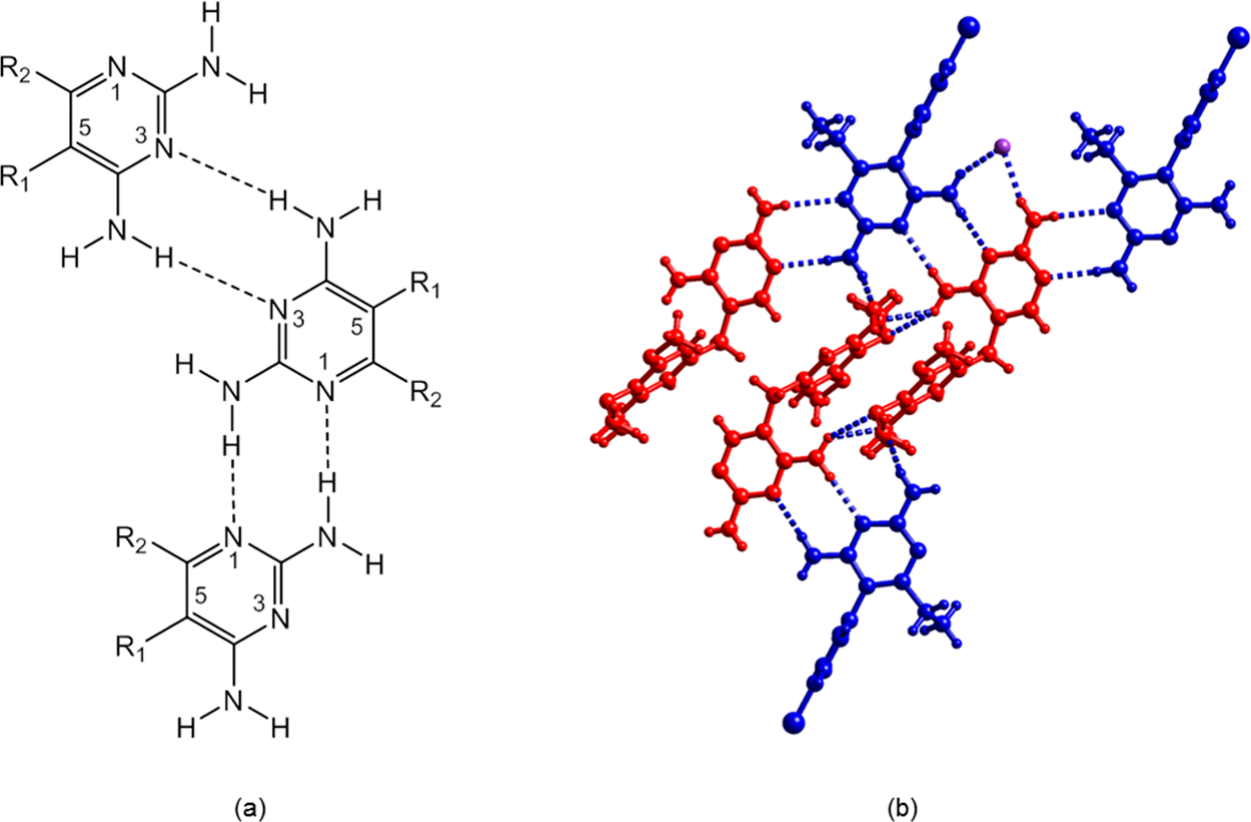
(a) Synthons in pyr and tmp. (b) H bonding motif in tmp.pyr·H_2_O. Red: tmp, blue: pyr, and purple: water molecule of crystallization. For clarity, only one component of the disordered water molecule of crystallization is shown.

The stable polymorphs of pyr and tmp have the same hydrogen-bonding motifs with pairwise H bonding at both amino-pyrimidine sites (Figure 2a).^40,41^ Pyr and tmp are therefore complementary coformers, and a cocrystal was indeed obtained from methanol. The crystal structure of the cocrystal of tmp and pyr is shown in Figure 2b. The asymmetric unit contains one tmp, one pyr, and one water molecule of crystallization. The *R*_2_^2^(8) homosynthons with pairs of C2–NH_2_···N1 and C4–NH_2_···N3 H bonds in the X-ray structures of pyr and tmp are replaced by the corresponding pyr···tmp heterosynthons. The second C4-amino proton of tmp forms a bifurcated H bond with two methoxy groups of an adjacent tmp. In addition, one methoxy group of tmp acts as a H bond acceptor for the C2-amino group of pyr. Overall, pyr and tmp are linked to an infinite 2D structure. The water molecule of crystallization is disordered over three positions and interacts with the C4-amino group of pyr and with the C2-amino group of tmp.

The cocrystal can also be prepared by ball-milling an equimolar mixture of tmp and pyr in the presence of a trace amount of ethanol. The match of the XRPD pattern of the milled sample and the theoretical pattern calculated from the single crystal data of tmp·pyr·H_2_O is shown in Figure S1. The DSC plot of the cocrystal (Figure S2) shows a dehydration endotherm at 124.1 °C (weight loss in the TGA 3.1%; calcd. 3.2%) and a melting endotherm at 195.9 °C. The latter temperature is lower than the melting points of tmp (200 °C) and pyr (233 °C).

### Interaction of Dicarboxylic Acid Coformers with the DA Binding Site of Tmp and Pyr

**Figure 3:**
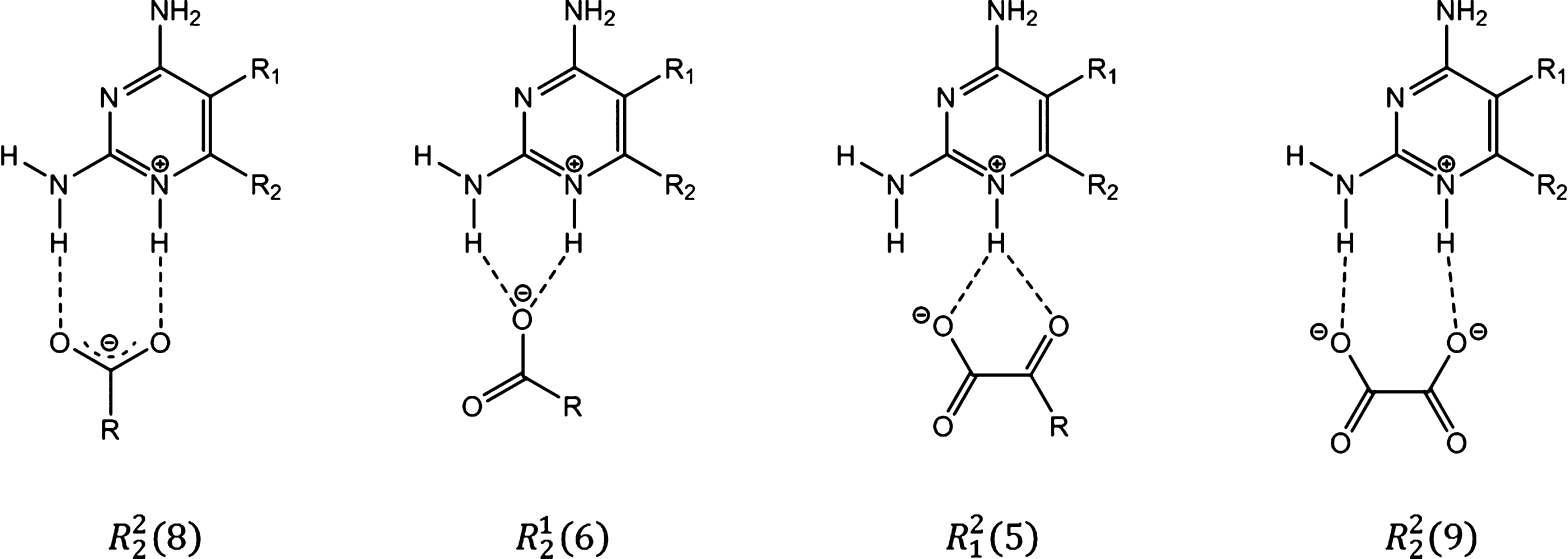
Heterosynthons in carboxylate salts of pyr and tmp.

A vast majority of crystal structures of cocrystals of tmp and pyr with a carboxylic acid coformer described in the literature show transfer of the carboxyl proton to the N1 nitrogen of pyr or tmp and the interaction of the carboxylate group with the N1–H^+^/C2–NH_2_ site via a pair of parallel H bonds creating the *R*_2_^2^(8) motif (Figure 3).^43−49^ In the following, we compare the H bonding interactions of the 2,4-diaminopyrimidine pharmacophore and carboxylic acid coformers for four carboxylic acids for which single crystal structures are available for both the tmp and the pyr system (either obtained in this study or published data). Contrary to what one might expect, differences in the main H bonding motif were observed between tmp/keto and pyr/keto and between tmp/ox and pyr/ox, while tmp/az and pyr/az and tmp/seb and pyr/seb were found to differ in the ionization states of the coformers (Table 1). (Pyr^+^)(keto^–^)^50^ and (pyr^+^)_2_(ox^–^)(ox^2–^)_0.5_·2CH_3_OH^51^ have the typical *R*_2_^2^(8) motif; however, this motif is absent in the corresponding tmp salts. For the sake of simplicity, we use the notation pyr·X/tmp·X for a cocrystal and pyr^+^X^–^/tmp^+^X^–^ for a salt with proton transfer from the coformer to the N1 nitrogen of pyr/tmp.

**Table 1:** Binding Sites and Tmp/Pyr-Coformer Synthons Observed in the Crystal Structures of Tmp and Pyr Cocrystals and Salts

	binding sites and synthons	
coformer	tmp	pyr
Keto	1:1; salt; DA site; *R*_1_^2^(5), *R*_2_^1^(6)^a^	1:1; salt; DA site; *R*_2_^2^(8)^b^
Ox	2:1; salt; DA site; *R*_1_^2^(5), *R*_2_^1^(6)^a^	2:1.5; salt; DA site; *R*_2_^2^(8)^c^
		2:1; salt; DA site; *R*_2_^2^(9)^a^
seb	2:1; salt; DA site; *R*_2_^2^(8)^a^	2:1; cocrystal; DA site; *R*_2_^2^(8)^a^
az	1:1; salt; DA site; *R*_2_^2^(8)^a^	1:1; salt; DA site; *R*_2_^2^(8)^a^
	1:1; cocrystal; DA and DAD site; *R*_2_^2^(8)–*R*_4_^2^(8)–*R*_2_^2^(8) rings^a^	
	2:1:1; ionic cocrystal; DA and DAD site; C–NH_2_···O^a^	
smz	1:1; cocrystal, DA site; *R*_2_^2^(8)^d^	1:1; cocrystal; DAD site; NH_2_···N/N···NH_sulfonamide_/NH_2_···O=S^a^
	1:2; cocrystal, DA site; *R*_2_^2^(8)^e^	

a This work.

b Reference (50).

c Reference (51).

d Reference (22).

e Reference (23).

**Figure 4:**
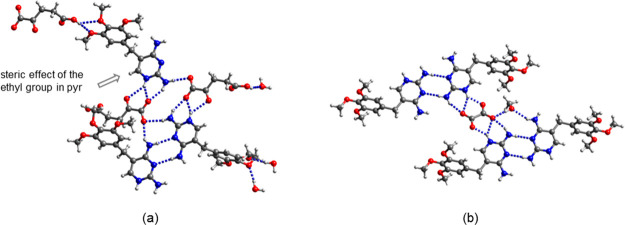
H bonding motif in (a) (tmp^+^)(keto^–^)·0.5H_2_O and (b) (tmp^+^)_2_(ox^2^)·2CH_3_OH.

*Tmp*/*keto*. The asymmetric unit of (tmp^+^)(keto^–^)·0.5H_2_O contains two crystallographically independent N1-protonated tmp^+^ cations, two keto^–^ monoanions, and a water molecule of crystallization. As expected, the carboxyl group next to the keto group is deprotonated and the proton transfer is confirmed by the C–O bond lengths that are equal within the standard deviations (C15–O4 1.235(2) Å, C15–O5 1.239(2) Å, C34–O12 1.238(2) Å, and C34–O13 1.232(2) Å) and by the increase of the angle at N1 from 114.87(9)° in neutral tmp^42^ to 119.6(2)/119.7(2)°. One of the carboxylate oxygens interacts with the N1–H^+^/C2–NH_2_ site in an *R*_2_^1^(6) motif (Figure 4a). The protonated N1 nitrogen acts as a bifurcated H bond donor to the 2-oxoacid site (graph set notation *R*_1_^2^(5)). The tmp^+^···keto^–^ ion pairs are connected through H bonding between the C2-amino group and the second carboxylate oxygen, giving rise to *R*_4_^4^(12) rings. There is also H bonding between the second carboxylate oxygen and the C4-amino group of another tmp^+^ cation. Neighboring cations form the *R*_2_^2^(8) homosynthon via pairs of C4–NH_2_···N3 H bonds. The protonated carboxyl group forms a bifurcated H bond with two of the methoxy groups of tmp^+^. The water molecule of crystallization interacts with the methoxy groups and the carboxyl group of keto^–^.

The DSC plot (Figure S3) shows a melting endotherm at 134.9 °C. A second, broad endotherm at 160–190 °C is accompanied by a weight loss in the TGA and is attributed to the decomposition of keto. The melting point of the salt is in the range between the melting points of the two coformers (tmp: 199–203 °C; keto: 113.5 °C).

*Tmp*/*ox and pyr*/*ox.* The *R*_2_^1^(6) and *R*_1_^2^(5) motifs are also present in (tmp^+^)_2_(ox^2–^)·2CH_3_OH (Figure 4b) and (tmp^+^)_2_(ox^2–^)·6.5H_2_O (Figure S4). In contrast to the C4–NH_2_···N3 *R*_2_^2^(8) homosynthon in (tmp^+^)_2_(ox^2–^)·6.5H_2_O, the tmp^+^ cations in (tmp^+^)_2_(ox^2–^)·2CH_3_OH interact with each other through the C2–NH_2_/N3 site, while the amino group at C4 donates a H bond to the solvent molecule of crystallization. The latter in turn forms a H bond with an oxalate oxygen, creating an *R*_4_^3^(10) motif.

Furthermore, we obtained single crystals of (pyr^+^)_2_(ox^2–^)·1.5H_2_O, which is a new stoichiomorph of the known oxalate salt of pyr.^51^ In this 2:1 salt, one oxygen of each carboxylate group is involved in a pair of NH···O H bonds (*R*_2_^2^(9), Figures 3 and S5).

The 2,4-diaminopyrimidine rings in pyr and tmp have almost the same p*K*_a_ value (7.16 and 7.34, respectively), indicating very similar electronic properties and H bonding propensity. The fact that the *R*_1_^2^(5)–*R*_2_^1^(6) H bonding pattern is only observed in tmp^+^ oxalates may be due to a steric hindrance effect of the ethyl substituent at C6 of pyr (Figure 4a).

Milling of tmp and ox in a 2:1 ratio in the presence of traces of methanol gave an XRPD pattern that is a good match with the theoretical pattern of (tmp^+^)_2_(ox^2–^)·2CH_3_OH (Figure S6). (Tmp^+^)_2_(ox^2–^)·6.5H_2_O and (pyr^+^)_2_(ox^2–^)·1.5H_2_O could not be obtained as phase-pure solids in bulk quantities, neither by milling nor by scaling up the crystallization experiment. Thermal analysis of (tmp^+^)_2_(ox^2–^)·2CH_3_OH (Figure S7) revealed the loss of the solvent molecules at 116.3 °C followed by two exothermic events at 161.9 and 172.6 °C and two endothermic events at 200.0 and 222.0 °C The latter peak has a shoulder at 235.6 °C. The exotherms are assigned to the formation of new phases of the desolvated salt. The endotherm at 200.0 °C may indicate the presence of “free” tmp (melting point 199–203 °C) and may suggest that the new phases are different stoichiomorphs. Melting of these phases gives the peak/shoulder at 222.0/235.6 °C. The TGA plot shows that melting is accompanied by the sublimation of ox (theoretical weight loss: 12.3%; observed weight loss: 11.9%).

**Figure 5:**
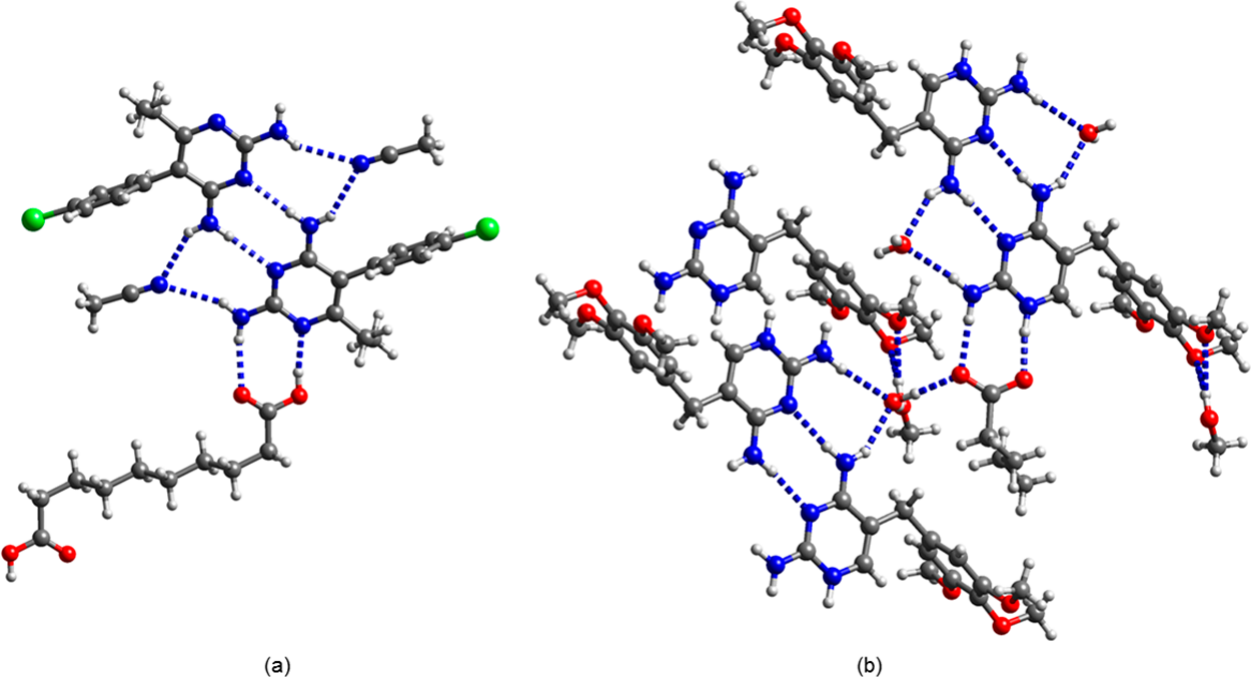
H bonding motif in (a) pyr.0.5seb·CH_3_CN and (b) (tmp^+^)_2_(seb^2–^)·2CH_3_OH·2H_2_O.

*Tmp*/*seb and pyr*/*seb.* Seb interacts with tmp and pyr via the typical pairwise H bonding with the C2–NH_2_/N1 site (*R*_2_^2^(8)). However, tmp and seb form a 2:1 salt of composition (tmp^+^)_2_(seb^2–^)·2CH_3_OH·2H_2_O, while no proton transfer takes place between pyr and seb and a cocrystal was obtained. Except for the position of the carboxyl proton, the main H bonding motifs are the same in both structures (Figure 5). The tmp^+^ ions are connected through a pair of H bonds involving the amino group at C4 and the ring nitrogen N3. CH_3_CN in pyr.0.5seb·CH_3_CN and water in (tmp^+^)_2_(seb^2–^)·2CH_3_OH·2H_2_O bridge the 2-amino and 4-amino groups on both sides of the pair of C4–NH_2_···N3 H bonds, creating an *R*_3_^2^(8) motif.

**Figure 6:**
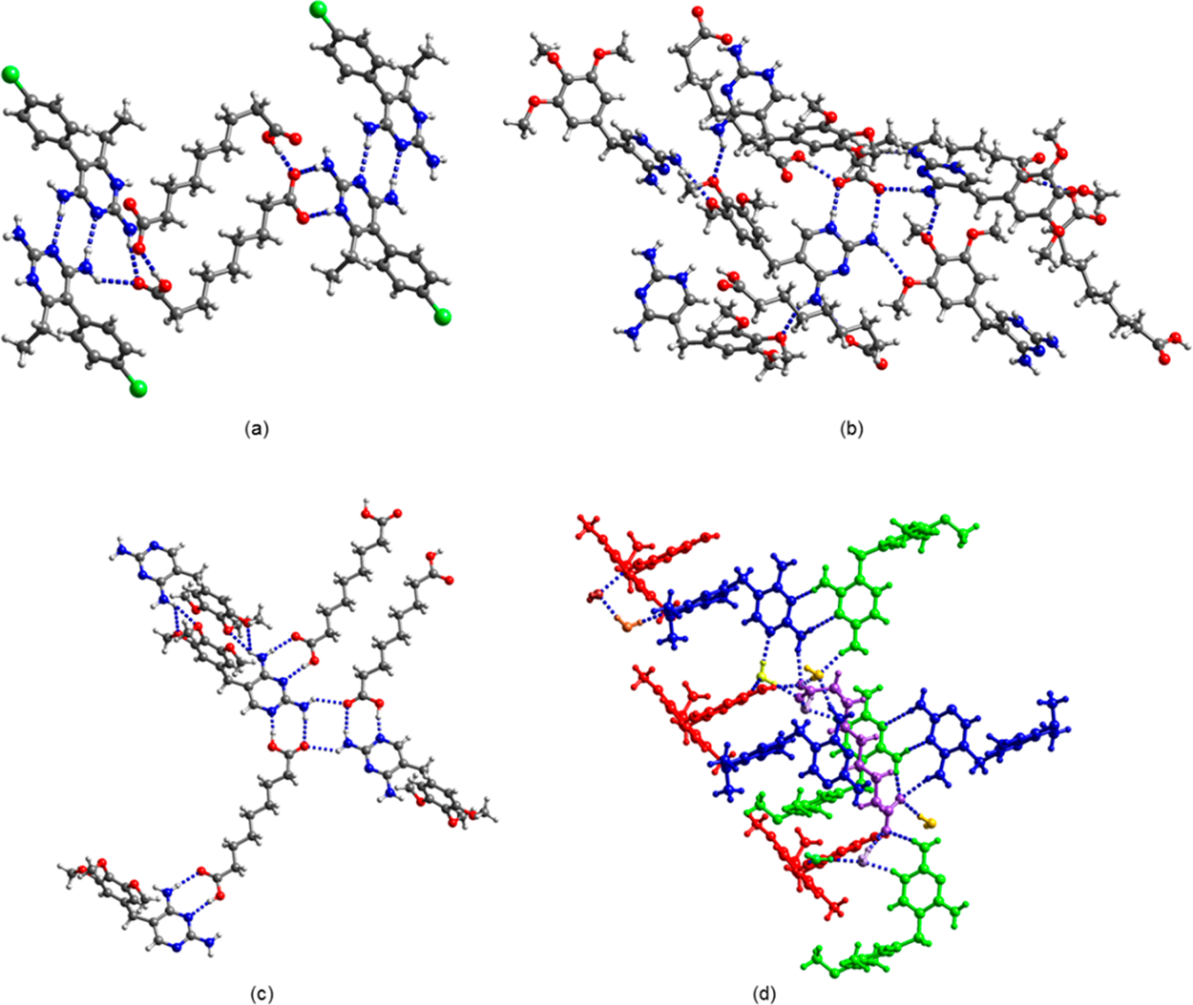
H bonding motif in (a) (pyr^+^)(az^–^) form I, (b) (tmp^+^)(az^–^), (c) tmp·az, and (d) (tmp^+^)_2_(az^2–^)·tmp·6H_2_O; blue: tmp, green: tmp^+^ A, red: tmp^+^ B, purple: az^2–^, and yellow and brown: water of crystallization.

*Tmp/az and pyr/az*. Differences in the interactions with tmp and pyr were also observed in the case of the coformer az. Pyr crystallized with az from acetonitrile as the 1:1 salt (pyr^+^)(az^–^) with the common *R*_2_^2^(8) heterosynthon (Figure 6a). Two az^–^ monoanions self-assemble into a 24-membered ring and pairs of C4–NH_2_···N3 H bonds connect the pyr^+^ cations to centrosymmetric dimers. H bonding between the carbonyl oxygen of the COOH group and the pyr^+^ dimers creates an *R*_3_^2^(8) motif. Co-sublimation of pyr and az gave a second polymorph, (pyr^+^)(az^–^) form II. Both forms have the same H bonding motifs, but differ in the dihedral angle between the pyrimidine and phenyl rings of tmp (82.9° in form I and 87.8° in form II). By contrast, in the case of tmp, a salt and a cocrystal were obtained, both containing the *R*_2_^2^(8) motif with the position of the proton differing (Figure 6b,c). The C–O bond lengths in az [C15–O4 1.214(3) Å; C15–O5 1.299(3) Å; C23–O6 1.206(3) Å; and C23–O7 1.319(3) Å] confirm that no proton transfer has taken place between the carboxylic acid and the N1 nitrogen in tmp·az. Both carboxyl groups of az form a pair of H bonds with tmp in the cocrystal, one COOH group interacts with the N1/C2–NH_2_ site and the other one interacts with the N3/C4–NH_2_ site. The carbonyl oxygen of the former also accepts a H bond from an adjacent C2-amino group, leading to a DADA array of quadruplex H bonding pattern or a motif of fused *R*_2_^2^(8)–*R*_4_^2^(8)–*R*_2_^2^(8) rings. One of the C4-amino protons forms a bifurcated H bond to two methoxy oxygens. Neither the salt nor the cocrystal contains the C4–NH_2_···N3 homosynthon.

The tmp·az single crystal was obtained from acetonitrile. A comparison of the XRPD pattern of the crystalline sample isolated from acetonitrile with the theoretical powder pattern of tmp·az confirmed that the single crystal structure was representative of the bulk composition (Figure S8). The single crystal of the (tmp^+^)(az^–^) salt was isolated from ethyl acetate. However, XRPD analysis of the bulk sample showed the pattern of the cocrystal, indicating that the salt is a minor side product or a transient form. Attempts to prepare bulk quantities of the salt by rapid solvent evaporation were unsuccessful.

A third form, the ionic cocrystal (tmp^+^)_2_(az^2–^)·tmp·6H_2_O, crystallized from methanol (Figure 6d). The asymmetric unit comprises two tmp^+^ cations (denoted A and B), one neutral tmp and one az^2–^ dianion and six water molecules of crystallization. In contrast to the 1:1 tmp·az cocrystal, (tmp^+^)_2_(az^2–^)·tmp·6H_2_O does not contain the C–NH_2_/N(H)···(H)OOC *R*_2_^2^(8) motif. The az^2–^ dianion participates in H bonding with the C2- and C4-amino groups of tmp, the C2-amino group of tmp^+^ A, and the C2-amino group of tmp^+^ B and with the water molecule of crystallization. Tmp^+^ A and tmp interact via a pair of H bonds between the N3/C4–NH_2_ site of the cation and the C2–NH_2_/N3 site of the neutral molecule. There is also extensive H bonding between the water of crystallization and the amino groups of tmp^+^ A, tmp^+^ B, and tmp, a methoxy group of tmp, and N1 of tmp.

Figure S9 shows the XRPD patterns after milling a 1:1 mixture of tmp and az in the presence of traces of ethanol, acetonitrile, and ethyl acetate. In all cases, the Bragg peaks of the cocrystal were observed. When a 3:1 mixture was milled in the presence of traces of H_2_O, the XRPD pattern of (tmp^+^)_2_(az^2–^)·tmp·6H_2_O was obtained (Figure S10). On slurrying in methanol, (tmp^+^)_2_(az^2–^)·tmp·6H_2_O converted to tmp·az (Figure S11). The DSC plot of (tmp^+^)_2_(az^2–^)·tmp·6H_2_O shows endotherms at 90.4, 155.3, and 185.8 °C (Figure S12a). The first endothermic event is accompanied by a 10.1% weight loss in the TA plot and can be assigned to the loss of the water of crystallization (calcd. 9.3%). The peak at 155.3 °C corresponds to the melting point of tmp·az (Figure S12b). When a sample of (tmp^+^)_2_(az^2–^)·tmp·6H_2_O was heated to 90 °C under vacuum for 6 h, XRPD analysis showed the pattern of free tmp and minor peaks that may be assigned to the cocrystal (Figure S13).

### Stable Form of Tmp/Az and Pyr/Az

The only form that was identified in cocrystallization experiments for pyr and az is the salt (pyr^+^)(az^–^). By contrast, solution crystallization of tmp/az suggests that the cocrystal is in the thermodynamically stable form, which was confirmed by slurry experiments. A slurry normally gives the thermodynamically stable product, and slurrying has been used in the literature to identify the most stable polymorph.^52,53^ No change in the XRPD pattern was observed, when the cocrystal was slurried for 48 h in methanol (Figure S14).

It has been reported for the ethionamide/salicylic acid system that proton transfer is not influenced by the nature of the solvent (polar/apolar; protic/aprotic) but that the crystallization of the salt/cocrystal polymorphs follows Ostwald’s rule of stages.^27^ Furthermore, we have previously shown that proton transfer can take place in prenucleation clusters in the absence of solvent,^54^ which further corroborates the lack of a solvent effect on the ionization state of the coformers in the solid state. The intermediate formation of the (tmp^+^)(az^–^) salt as a metastable transient form may be the reason why it was not possible to isolate bulk quantities.

The p*K*_a_ values of the N1 nitrogen of pyr and for the deprotonation of the first carboxyl group of az are 7.34 and 4.15, respectively, so that salt formation for the pyr/az system is in line with the Δp*K*_a_ rule that states that a salt is usually obtained when the difference in the p*K*_a_ of the two coformers is greater than 4, while a Δp*K*_a_ of <0 results in a cocrystal.^55,56^ In the Δp*K*_a_ range 0 < p*K*_a_ < 4, there is a salt-cocrystal continuum and a prediction of salt or cocrystal formation is not possible. The p*K*_a_ value of tmp is 7.16 and is thus very close to that of pyr. Cruz-Cabeza has developed a model that estimates the probability for cocrystal and salt formation as^57^
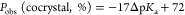

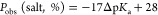


Using these equations, there is a 21% probability of tmp and az crystallizing as a cocrystal and a 79% probability for salt formation. The position of the proton is determined by the packing and the resulting lattice energy. While the amount of proton transfer can be affected by the crystalline environment, Δp*K*_a_ represents a useful predictive tool outside the continuum region.^56^ Childs et al. compared the unintended application of p*K*_a_ values to predict the ionization state in the solid state with the use of van der Waals radii, which were originally intended for the calculation of molecular volume but are now routinely used to estimate intermolecular distances in adjacent molecules.^56^ Tmp·az and (tmp^+^)(az^–^) are a rare example of cocrystal–salt polymorphism. The protonation state of the coformers in multicomponent crystals can vary for different stoichiometries or solvates.^58−60^ However, only a small number of systems have been reported, where a salt or a cocrystal of the same composition and stoichiometry is obtained depending on the crystallization conditions; smz/saccharine (smz/sac),^28,29^ four amino acids/tartaric acid of which β-alanine/tartaric acid (bal/tar), has been examined in detail,^61,62^ ethionamide/salicylic acid (eth/sal),^27^ and a range of haloaniline/3,5-dinitrobenzoic acid systems.^30^ The position of the proton may also be temperature-dependent.^63,64^ Bal/tar^62^ and smz/sac^28,29^ crystallize stochastically as salts or cocrystals from the same solution. Concomitant growth of salt and cocrystal polymorphs has also been observed for haloaniline/3,5-dinitrobenzoic acid,^30^ and a metastable salt was identified as a transient form to the polymorphs of the 2:1 isonicotinamide–citric acid cocrystal.^65^ The stable form, salt or cocrystal, was deduced in each case using crystal slurries and other methods, and in the case of the haloaniline/3,5-dinitrobenzoic acid series, the energy differences per formula unit between the salt and cocrystal were calculated by periodic boundary DFT methods using the program VASP. In that series of compounds, the salts were more stable than the cocrystal forms without exception. In the case of 4-bromo-2-methylaniline/3,5-dinitrobenzoic acid (brma/dnb), the salt was reported to be 8.5 kJ/mol more stable than the cocrystal.

We have calculated the energy differences between the salt and the cocrystal for tmp/az and for the other systems described in the literature (Table 2), and in every case, periodic DFT calculations predict the stable form found by the experiment. Positive Δ*E*_(sa-cc)_ values indicate that the cocrystal is more stable than the salt.

**Table 2:** Energy Differences Calculated for Some Systems, Which Have Stoichiometry B·HA and BH^+^A^–^

compound	Δp*K*_a_^a^^,^^b^	Δ*E*_(sa-cc_)^c^	stable form	density_salt_	density_cocrystal_/g cm^–3^
bal/tar	0.52	68.6	cocrystal	1.655	1.626
smz/sac	0.80	7.4	cocrystal	1.450	1.569
brma/dnb	0.89	–9.3	salt	1.717	1.730
eth/sal	2.0	–6.7	salt	1.332	1.298
tmp/az	3.0	12.2	cocrystal	1.223	1.307

a Δp*K*_a =_ p*K*_a_(B) – p*K*_a_(HA).

b p*K*_a_ values taken from refs (67) and (68).

c Δ*E*_(sa-cc)_ = (*E*_(salt)_/*Z* – *E*_(cocrystal)_/*Z*)/kJ mol^–1^.

Crystal density is an indication of the efficiency of crystal packing and can be used as an indication of crystal stability. For example, crystal density has been used to select the most likely candidates in crystal structure prediction software.^66,67^ In Table 2, crystal density correctly predicts the stable form in three out of the five examples, with bal/tar and brma/dnb having stable forms with lower densities than their metastable forms. Thus, at least in the examples in Table 2, crystal density is less effective than periodic DFT calculations in predicting the stability of crystal structures.

There is also one interesting difference between tmp/az and the other systems in Table 2 in that it was necessary to apply nonlinear distance restraints to the O–H groups H-bonded to nitrogen to prevent proton transfer. This of course does not imply that the crystallized salt is more stable than the cocrystal, rather this proton transfer suggests that there may be another salt form, which would crystallize in space group P-1 if suitable conditions for its isolation could be found.

Noteworthily, among the salt-cocrystal polymorph pairs listed in Table 2, tmp/az has the largest Δp*K*_a_ difference between the two coformers. To understand the higher stability of the tmp·az cocrystal and the difference to the pyr/az system, we have examined the packing of the tmp salt, tmp cocrystal, and pyr salt. The densities of (tmp^+^)(az^–^), tmp·az, and (pyr^+^)(az^–^) are 1.223, 1.307, and 1.252 g cm^–3^, respectively, that is, the density of (pyr^+^)(az^–^) is intermediate between those of tmp·az and (tmp^+^)(az^–^). (Pyr^+^)(az^–^) contains Cl and would be expected to have a higher density than either of the tmp/az forms. Using the Void program in the Oscail package,^69^ the packing indices for tmp·az, (tmp^+^)(az^–^), and (pyr^+^)(az^–^) were calculated as 68.5, 70.5, and 63.7%. The Void program also finds a 3.3% void, which is large enough to insert one water molecule into the (pyr^+^)(az^–^) structure. The program did not find any void in either of the tmp/az structures. The void locations in (pyr^+^)(az^–^) are shown in the packing view down the *c* axis (Figure S15). It is possible that the restriction imposed by the biphenyl linkage in the pyr molecule, which prevents the ring packing in a more efficient flat geometry, leads to a lower packing efficiency than is observed in the tmp/az structures, where the flexible nature of tmp may aid efficient packing. The observation of a salt rather than a cocrystal for pyr/az may be due to an extra lattice energy component provided by the ionic attractions in the salt. It is worthy to note that in tmp·az, both pyrimidine-N/NH_2_ sites form a pair of H bonds with az-COOH, while in (tmp^+^)(az^–^), only the N1/C2–NH_2_ site forms the *R*_2_^2^(8) motif and N3 is not involved in H bonding.

### Different Interactions of Tmp and Pyr with Sulfa Drugs

**Figure 7:**
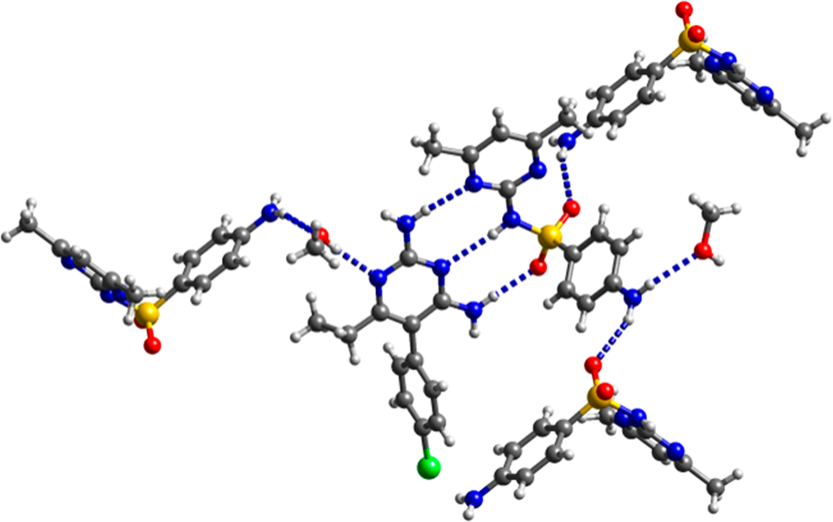
H bonding motif in pyr.smz·CH_3_OH. Only one component of the disordered ethyl group of pyr is shown.

The cocrystallization of tmp with the sulfa drugs sulfamethoxazole (smx), sulfametrole (smt), sulfamethoxypyridazine (smp), and smz is described in the literature, and the X-ray structures of the salts (tmp^+^)(smx^–^),^19^ (tmp^+^)(smt^–^),^20^ and (tmp^+^)(smp^–^)·1.5H_2_O^21^ and of the cocrystals tmp·smp,^21^ tmp·smz·CH_3_OH,^22^ and tmp·2smz·H_2_O^23^ have been reported. The sulfa drugs have an N(heterocycle)=CH–NH(sulfonamide) functionality, and in all cases, the heterocyclic nitrogen and the sulfonamide nitrogen form a pair of H bonds with the C2–NH_2_/N1 site of tmp (*R*_2_^2^(8)). Interestingly, we obtained the single crystal structure of the smz cocrystal of pyr that showed binding of the sulfa drug via the DAD site of pyr (Figure 7). In pyr·smz·CH_3_OH, one of the sulfonyl oxygens, the sulfonamide nitrogen and the ring nitrogen of smz form H bonds with the amino group at C4, the N3 nitrogen, and the amino group at C2 of pyr. The second sulfonyl oxygen accepts a H bond from the amino group of an adjacent smz. The methanol molecule of crystallization participates in H bonding with N1 of pyr and the amino group of smz. Again, the steric hindrance of the ethyl substituent on the carbon adjacent to N1 may be the reason why smz does not bind to the C2–NH_2_/N1 site as is the case in the tmp·smz cocrystal. It appears that the methanol of crystallization stabilizes the structure by filling the voids resulting from the formation of the ADA···DAD synthon.

The DSC plot of pyr·smz (Figure S16) shows an endotherm at 111.5 °C followed by an exotherm at 136.2 °C that can be assigned to the loss of the hydrogen-bonded methanol of recrystallization and the structural rearrangement of the desolvated form. The evaporation of methanol is confirmed by the weight loss in the TGA plot. Two other endothermic events occur at 186.1 and 214.7 °C, which is close to the melting points of smz and pyr.

### Ternary Cocrystals of Tmp

The observation that smz can act as an ADA coformer at the C2–NH_2_/N3/C4–NH_2_ site of the 2,4-diaminopyrimidine ring in pyr·smz·CH_3_OH prompted us to explore the formation of ternary cocrystals of tmp/pyr, a sulfadrug, and a carboxylic acid. We expected that in such ternary mixtures, the carboxylic acid would have the highest affinity for the C2–NH_2_/N1 site of tmp/pyr: cocrystals of sulfa drugs and carboxylic acids are rather scarce. Very recently, it is has been stated in the literature that “the chance to form a successful cocrystal containing the sulfa drug and benzoic acid or one of its derivatives is low”.^70^ In the same article, a cocrystal screen of sulfathiazole (stz), sulfamethoxazole, and sulfapyridine with a range of coformers including carboxylic acids was reported. Out of the 30 sulfa drug–carboxylic acid combinations tested, only three were successful. These include the 3,5-dinitrobenzoate salt of stz, the sulfamethoxazole-3,5-dinitrobenzoic acid cocrystal, and the 2-chloro-4-nitrobenzoate salt of sulfapyridine. A slightly higher success rate was reported for a smz cocrystal screen that led to the structural characterization of 7 smz cocrystals with aliphatic and aromatic carboxylic acids. In all cases, the carboxylic acid formed a pair of H bonds with the sulfonamide/N(heterocycle) site of either the amidine or the imidine tautomer of smz.^71^ This *R*_2_^2^(8) motif was also observed in the crystal structures of salts and cocrystals with benzoic acid,^72^ salicylic acid,^73^ anthranilic acid, 4-aminobenzoic acid,^74^ 4-aminosalicylic acid, acetylsalicylic acid,^75^ 2-nitrobenzoic acid,^76^ 4-nitrobenzoic acid,^77^ 2,4-dinitrobenzoic acid, indole-2-carboxylic acid,^78^ 4-chlorobenzoic acid,^79^ and 5-nitrosalicyclic acid.^80^ By contrast, stz/3,5-dinitrobenzoic acid, sulfamethoxazole/3,5-dinitrobenzoic acid, and sulfapyridine/2-chloro-4-nitrobenzoic acid all have different synthons.^70^ In the stz·glutaric acid^81^ and stz·4-nitrobenzoic acid^82^ cocrystals, stz and the carboxylic acid both form homodimers. In stz·glutaric acid, the dimers are linked via a bifurcated H bond to the amino group and sulfonyl oxygen of two adjacent stz molecules. In stz·4-nitrobenzoic acid, the homodimers interact with each other via π–π contacts between the thiazole and nitrobenzene rings.

The carboxylic acid and sulfonamide combinations used in the screening study are listed in Tables S6 and S7. The isolation of a ternary cocrystal was only successful in the case of stz and tmp, and instead of binding to the DAD site of tmp, a new ternary synthon was observed with stz interacting with the tmp^+^···carboxylate dimer via S=O···NH_2_ and NH···O H bonds. It is worthy to note that while in several cases, the binary pyr^+^/tmp^+^ carboxylate salt crystallized from solution in the screening study for ternary cocrystals, no binary sulfa drug/carboxylic acid cocrystal was obtained, which further confirms the low affinity of the carboxyl group for the sulfonamide/N-heterocycle binding site.

**Figure 8:**
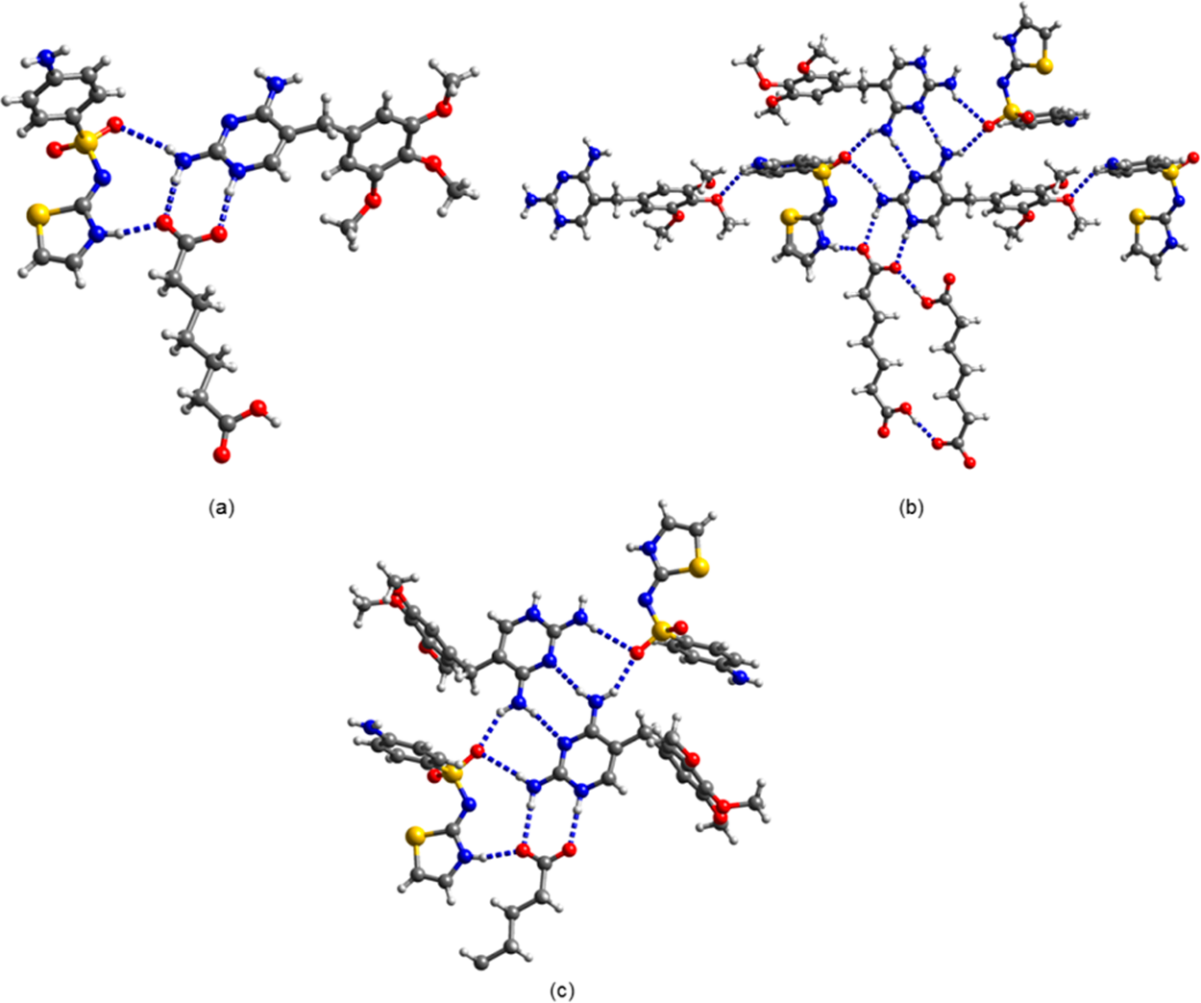
(a) Ternary synthon in (tmp^+^)(pim^–^)·stz. (b) Extended H bonding motif in (tmp^+^)(pim^–^)·stz. (c) H bonding motif in (tmp^+^)_2_(seb^2–^)·stz·2H_2_O·C_3_H_6_O. Water and solvent molecules of crystallization are omitted for clarity.

The H bonding patterns of the two ternary ionic cocrystals obtained, (tmp^+^)(pim^–^)·stz and (tmp^+^)_2_(seb^2–^)·stz·2H_2_O·C_3_H_6_O, are shown in Figure 8. In both structures, stz exists in its imido tautomeric form and forms the same ternary synthon with tmp^+^ and the carboxylate group. H bonding between a sulfonyl oxygen and the amino group at C2 and between the proton on the heterocyclic ring nitrogen and a carboxylate oxygen connect the sulfa drug to the tmp^+^···carboxylate synthon, leading to a motif of fused *R*_3_^2^(10) and *R*_2_^2^(8) rings. Furthermore, both structures have the C4–NH_2_···N3 homosynthon. Additional hydrogen bonding of the C2- and C4-amino groups with S=O creates *R*_3_^2^(8) rings. In (tmp^+^)(pim^–^)·stz, two pim monoanions dimerize into a 20-membered ring, while the seb^2–^ dianion in (tmp^+^)_2_(seb^2–^)·stz·2H_2_O·C_3_H_6_O lies on an inversion center, so that both carboxylate groups interact with stz and tmp^+^. It is interesting that in contrast to (tmp^+^)(pim^–^)·stz, no proton transfer from the carboxyl group to the 2,4-diaminopyrimidine nitrogen takes place when tmp and pim cocrystallize to give the binary cocrystal tmp·pim·0.5CH_3_CN (Figure S17).

Milling experiments were carried out to investigate if the two ternary cocrystals could also be prepared mechanochemically. The XRPD patterns of the milled tmp/stz/seb sample (1:1:1 molar ratio) showed a new set of peaks and unreacted stz (Figure S18). The new peaks are identical to the pattern obtained when a binary 1:1 mixture of tmp and seb is milled. In the case of the 1:1:1 tmp/stz/pim sample, the XRPD pattern matched the theoretical pattern of (tmp^+^)(pim^–^)·stz (Figure S19), indicating that the ternary cocrystal can also be prepared mechanochemically.

## Conclusions

Despite the identical H bonding functionality and almost identical p*K*_a_ values, the 2,4-diaminopyrimidine groups in tmp and pyr do not necessarily form the same H bonding motif with a given coformer. Crystal structures have been obtained, where the same coformer binds to the N1/C2–NH_2_ site of tmp (pairwise H bonding) but to the C2–NH_2_/N3/C4–NH_2_ site of pyr (three parallel H bonds) or where the coformer interacts at the same site but gives H bonding patterns with different graph set notations, specifically fused *R*_2_^1^(6) and *R*_1_^2^(5) rings versus the *R*_2_^2^(8) motif in carboxylates. These examples demonstrated the significant effect of the ethyl substituent on the C6 carbon of pyr. The tmp/az system shows salt-cocrystal polymorphism with the salt, presenting a transient metastable form, contrary to what one would expect on the basis of the Δp*K*_a_ rule, the Coulomb attraction contribution to the lattice energy, and the calculated packing indices of the salt and cocrystal forms. In ternary cocrystals, two coformers usually bind to two different functional groups or binding sites of the third component. Instead, all three components are connected in an *R*_3_^2^(10) motif, generating a unique ternary motif in (tmp^+^)(pim^–^)·stz and (tmp^+^)_2_(seb^2–^)·stz·2H_2_O·C_3_H_6_O. We have reported three-component ionic cocrystals of pyr with ADA- and AD coformers in a previous paper.^18^ The ADA- and AD-coformers bound to different pyr molecules in the crystal lattice. Apparently, the simultaneous interaction of two different coformers at both binding sites of the 2,4-diaminopyrimidine ring is not favorable.

## Supporting Information Available

Details on the ball-milling and crystallization experiments, crystal data, H bonding interactions, X-ray structures of (tmp^+^)_2_(ox^2–^)·6.5H_2_O, (pyr^+^)_2_(ox^2–^)·1.5H_2_O, and tmp·pim·0.5CH_3_CN, XRPD patterns, and DSC and TGA plots (PDF)

## Accession Codes

CCDC 2129639–2129654 contain the supplementary crystallographic data for this paper. These data can be obtained free of charge via www.ccdc.cam.ac.uk/data_request/cif, or by emailing data_request@ccdc.cam.ac.uk, or by contacting The Cambridge Crystallographic Data Centre, 12 Union Road, Cambridge CB2 1EZ, UK; fax: +44 1223 336033.
