# Supplementary material for: Differences in Coformer Interactions of the 2,4-Diaminopyrimidines Pyrimethamine and Trimethoprim
Source: Cryst Growth Des. 2022 Apr 8;22(5):3163–73. doi: 10.1021/acs.cgd.2c00035 (PMC9073935; doi:10.1021/acs.cgd.2c00035)
Supplement: Supplementary file 1 — cg2c00035_si_001.pdf [file cg2c00035_si_001.pdf]

Differences in Coformer Interactions of the 2,4-Diaminopyrimidines Pyrimethamine and Trimethoprim

Lamis Alaa Eldin Refat,[1,2] Ciaran O'Malley,[1] John M. Simmie,[1] Patrick McArdle,[1,*] and Andrea Erxleben[1,2,*]

[1] School of Chemistry, National University of Ireland Galway, Galway, H91TK33, Ireland

[2] Synthesis and Solid State Pharmaceutical Centre (SSPC), Limerick, V94 T9PX, Ireland

*Corresponding author email address: andrea.erxleben@nuigalway.ie (AE); p.mcardle@nuigalway.ie (PM)

Supporting Information

Table S1. Sample compositions in the ball-milling experiments.

tmp	coformer 1	Coformer 2	Solvent	Result
50.0 mg	42.8 mg pyr	--	C_2H_5OH	tmp·pyr·H_2O
50.0 mg	15.5 mg ox	--	CH_3OH	$(tmp^+)_2ox^{2-}\cdot 2CH_3OH$
50.0 mg	25.1 mg keto	--	C_2H_5OH	new pattern
50.0 mg	32.4 mg az	--	C_2H_5OH	tmp·az
50.0 mg	32.4 mg az		CH_3CN	tmp·az
50.0 mg	32.4 mg az		$CH_3C(=O)OC_2H_5$	tmp·az
50.0 mg	10.8 mg az		H_2O	$(tmp^+)_2(az^{2-})\cdot tmp\cdot 6H_2O$
50.0 mg	28.0 mg pim	--	CH_3CN	new pattern
50.0 mg	34.8 mg seb	--	CH_3OH	new pattern
50.0 mg	47.7 mg stz	--	C_2H_5OH	new pattern
40.0 mg	27.8 mg seb	38.2 mg stz	$CH_3C(=O)CH_3$	new pattern + stz
40.0 mg	20.1 mg pim	38.2 mg stz	CH_3CN	$(tmp^+)(pim^-)\cdot stz$

Table S2. Crystal data of tmp·pyr·H_2O, $(tmp^+)(keto^-)\cdot 0.5H_2O$, pyr·smz·$CH_3OH$, $(pyr^+)_2(ox^{2-})\cdot 1.5H_2O$, and $(tmp^+)_2(ox^{2-})\cdot 2CH_3OH$.

	tmp·pyr·H_2O	$(tmp^+)(keto^-)\cdot 0.5H_2O$	pyr·smz·CH_3OH	$(pyr^+)_2(ox^{2-})\cdot 1.5H_2O$	$(tmp^+)_2(ox^{2-})\cdot 2CH_3OH$
Formula	$C_{26}H_{31}ClN_8O_4$	$C_{38}H_{50}N_8O_{17}$	$C_{25}H_{31}ClNO_3S$	$C_{52}H_{60}Cl_4N_{16}O_{11}$	$C_{16}H_{23}N_4O_6$
M_r	555.04	890.86	559.09	1226.96	367.38
Crystal color and habit	colorless plate	colorless plate	colorless needle	colorless plate	colorless block
Crystal size (mm)	0.40 x 0.35 x 0.10	0.70 x 0.40 x 0.05	0.80 x 0.05 x 0.05	0.80 x 0.20 x 0.05	0.80 x 0.65 x 0.45
Crystal system	triclinic	triclinic	monoclinic	orthorhombic	monoclinic
Space group	P-1	P-1	$P2_1/c$	Pnna	C2/c
a [Å]	8.9793(7)	10.6362(9)	7.2742(8)	18.7158(13)	22.0031(12)
b [Å]	10.6391(7)	11.4197(6)	15.981(2)	28.731(3)	9.5688(7)
c [Å]	16.1082(14)	18.5569(10)	24.343(3)	11.3639(7)	17.8913(12)
α [°]	95.436(6)	94.980(4)	90	90	90
β [°]	99.651(7)	104.597(6)	91.893(10)	90	105.903(6)
γ [°]	100.694(6)	103.891(6)	90	90	90
V [Å^3]	1478.1(2)	2091.2(2)	2828.3(6)	6110.7(8)	3622.7(4)
Z	2	2	4	4	8
D_{calc} (g cm^{-3})	1.247	1.415	1.313	1.334	1.347
Temperature (K)	297.7(1)	296.6(8)	296.1(6)	298.0(2)	296.7(3)
No. measd. reflections	10962	18114	20695	15263	8144
No. unique refl. (R_{int})	5394 (0.0656)	9644 (0.0393)	5164 (0.1684)	7145 (0.0605)	4203 (0.0198)
No. obs. reflections	2410	6000	2004	3007	3146
Final R_1, wR_2 (obs. refl.)	0.0678, 0.1580	0.0540, 0.1134	0.0882, 0.1656	0.0652, 0.1442	0.0469, 0.1257
Goodness-of-fit (obs. refl.)	0.957	0.995	0.909	0.942	1.025

Table S3. Crystal data of $(tmp^+)_2(ox^{2-})\cdot 6.5H_2O$, $(pyr^+)(az^-)$ form I and II, tmp·az, $(tmp^+)(az^-)$, and $(tmp^+)_2(az^{2-})\cdot tmp\cdot 6H_2O$.

	$(tmp^+)_2(ox^{2-})\cdot 6.5H_2O$	$(pyr^+)(az^-)$ form I	$(pyr^+)(az^-)$ form II	tmp·az	$(tmp^+)(az^-)$	$(tmp^+)_2(az^{2-})\cdot tmp\cdot 6H_2O$
Formula	$C_{30}H_{42}N_8O_{16.50}$	$C_{21}H_{29}ClN_4O4$	C_{21} H_{29} Cl N_4 O_4	$C_{23}H_{34}N_4O_7$	$C_{23}H_{34}N_4O_7$	$C_{51}H_{82}N_{12}O_{19}$
M_r	778.71	436.93	436.93	478.54	478.54	1167.28
Crystal color and habit	colorless plate	colorless block	colorless plate	colorless block	colorless plate	colorless block
Crystal size (mm)	0.70 x 0.30 x 0.05	0.85 x 0.65 x 0.40	0.60 x 0.40 x 0.10	0.40 x 0.20 x 0.10	0.70 x 0.60 x 0.10	0.65 x 0.30 x 0.10
Crystal system	triclinic	monoclinic	monoclinic	triclinic	orthorhombic	triclinic
Space group	P-1	$P2_1/c$	$P2_1/c$	P-1	Pbca	P-1
a [Å]	5.1132(4)	6.8146(3)	6.7423(3)	8.0656(7)	13.2886(7)	10.8103(4)
b [Å]	10.7995(7)	12.8847(7)	26.3885(13)	8.1821(8)	17.3385(13)	11.2043(5)
c [Å]	18.1645(16)	26.4111(13)	13.0314(8)	18.5783(17)	22.5624(13)	25.4525(11)
α [°]	79.470(7)	90	90	82.951(8)	90	89.581(4)
β [°]	83.062(6)	94.738(4)	91.059(6)	87.702(7)	90	88.914(3)
γ [°]	87.026(6)	90	90	88.513(7)	90	69.910(4)
V [Å^3]	978.47(13)	2311.1(2)	2318.1(2)	1215.5(2)	5198.5(6)	2894.8(2)
Z	1	4	4	2	8	2
D_{calc} (g cm^{-3})	1.322	1.256	1.252	1.307	1.223	1.339
Temperature	293.0(2)	297.0(1)	293.0(2)	296.0(7)	298.8(2)	297.0(1)
No. measd. reflections	7765	19649	18241	10536	15757	28638
No. unique refl. (R_{int})	4493 (0.0327)	5541 (0.0233)	5610 (0.0439)	5621 (0.0506)	6133(0.0417)	13442 (0.0364)
No. obs. reflections	1923	4096	2979	2581	2721	8404
Final R_1, wR_2 (obs. refl.)	0.0860, 0.2361	0.0593, 0.1654	0.0691, 0.1686	0.0591, 0.1093	0.0736, 0.1955	0.0561, 0.1192
Goodness-of-fit (obs. refl.)	0.975	1.001	0.992	0.914	0.976	0.995

Table S4. Crystal data of pyr·0.5seb·CH_3CN, $(tmp^+)_2(seb^{2-})$·2CH_3OH·2H_2O, tmp·pim·0.5CH_3CN, $(tmp^+)(pim^-)$·stz, and $(tmp^+)_2(seb^{2-})$·2stz·2H_2O·C_3H_6O.

	pyr·0.5seb·CH_3CN	$(tmp^+)_2(seb^{2-})$·2CH_3OH·2H_2O	tmp·pim·0.5CH_3CN	$(tmp^+)(pim^-)$·stz	$(tmp^+)_2(seb^{2-})$·2stz·2H_2O·C_3H_6O
Formula	$C_{19}H_{25}ClN_5O_2$	$C_{20}H_{33}N_4O_7$	$C_{44}H_{63}N_9O_{14}$	$C_{30}H_{39}N_7O_9S_2$	$C_{31}H_{44}N_7O_9S_2$
M_r	390.89	441.50	942.03	705.80	722.85
Crystal color and habit	colorless plate	colorless block	colorless block	colorless plate	colorless block
Crystal size (mm)	0.55 x 0.70 x 0.20	0.70 x 0.75 x 0.40	0.75 x 0.30 x 0.25	0.70 x 0.35 x 0.50	0.40 x 0.40 x 0.20
Crystal system	triclinic	triclinic	triclinic	monoclinic	triclinic
Space group	P-1	P-1	P-1	$P2_1/c$	P-1
a [Å]	8.6449(9)	10.8179(6)	8.5625(6)	18.5255(12)	9.1280(4)
b [Å]	9.1396(9)	10.8905(5)	15.7389(9)	22.4705(12)	10.6303(4)
c [Å]	14.5579(14)	11.5791(6)	19.5579(11)	8.2972(4)	19.8558(8)
α [°]	102.444(8)	88.766	101.305(5)	90	86.004(3)
β [°]	102.766(9)	74.291	97.033(5)	95.497(5)	84.068(4)
γ [°]	103.590(9)	64.891	105.581(5)	90	67.697(4)
V [Å^3]	1046.11(19)	1182.27(12)	2446.4(3)	3438.1(3)	1772.02(13)
Z	2	2	2	4	2
D_{calc} (g cm^{-3})	1.241	1.240	1.279	1.364	1.355
Temperature	298.2(4)	299.8(7)	299.1(6)	297.8(9)	149.9(1)
No. measd. reflections	9324	10516	22126	30141	15632
No. unique refl. (R_{int})	4858 (0.0289)	5423 (0.0242)	11295 (0.0215)	8366 (0.0569)	8258 (0.0263)
No. obs. reflections	2817	3358	6455	4901	6561
Final R_1, wR_2 (obs. refl.)	0.0592, 0.1525	0.0527, 0.1328	0.0668, 0.2168	0.0607, 0.1194	0.0425, 0.1001
Goodness-of-fit (obs. refl	0.980	0.950	0.912	1.020	0.993

Table S5. H bonding interactions in tmp·pyr·H_2O, $(tmp^+)(keto^-)\cdot 0.5H_2O$, pyr·smz·$CH_3OH$, $(pyr^+)_2(ox^{2-})\cdot 1.5H_2O$, $(tmp^+)_2(ox^{2-})\cdot 2CH_3OH$, $(tmp^+)_2(ox^{2-})\cdot 6.5H_2O$, $(pyr^+)(az^-)$ form I and II, tmp·az, $(tmp^+)(az^-)$, $(tmp^+)_2(az^{2-})\cdot tmp\cdot 6H_2O$, pyr·0.5seb·$CH_3CN$, $(tmp^+)_2(seb^{2-})\cdot 2CH_3OH\cdot 2H_2O$, tmp·pim·0.5$CH_3CN$, $(tmp^+)(pim^-)\cdot stz$ and $(tmp^+)_2(seb^{2-})\cdot 2stz\cdot 2H_2O\cdot C_3H_6O$.

	**D-H···A**	**d(D···A) (Å)**	**∠(DHA) (°)**	**symmetry code**
tmp·pyr·H_2O	N(3)-H(3A)...N(5)	3.128(4)	150.1	x,y-1,z
	N(3)-H(3B)...O(4A)	3.082(12)	157.2	
	N(3)-H(3B)...O(4C)	3.254(10)	143.3	
	N(8)-H(8A)...N(1)	3.022(5)	166.9	
	N(8)-H(8B)...O(4A)	3.067(10)	133.5	
	N(8)-H(8B)...O(4C)	3.007(8)	139.5	
	C(21)-H(21)...N(2)	3.411(5)	161.1	-x+1,-y,-z+1
	N(4)-H(1N4)...O(1)	3.084(4)	124(4)	-x+1,-y+1,-z+2
	N(4)-H(1N4)...O(2)	3.241(4)	137(4)	-x+1,-y+1,-z+2
	N(4)-H(2N4)...N(7)	3.122(5)	178(5)	
	N(6)-H(1N6)...O(1)	3.035(4)	155(4)	-x+1,-y+1,-z+2
	N(6)-H(2N6)...N(2)	2.988(4)	171(4)	x,y+1,z
$(tmp^+)(keto^-)\cdot 0.5H_2O$	N(3)-H(1N3)...N(6)	3.031(2)	176(2)	x,y-1,z
	N(3)-H(2N3)...O(13)	2.757(2)	134.6(18)	x,y-1,z
	N(4)-H(1N4)...O(4)	2.979(2)	167(2)	
	N(4)-H(2N4)...O(12)	2.797(2)	152(2)	
	N(1)-H(1N1)...O(12)	2.698(2)	149(2)	
	N(1)-H(1N1)...O(14)	3.038(2)	133.3(19)	
	N(5)-H(1N5)...O(12)	3.071(2)	117.8(17)	
	N(5)-H(1N5)...O(13)	2.878(2)	172(2)	
	N(5)-H(2N5)...O(5)	2.813(2)	147.6(18)	
	N(8)-H(1N8)...N(2)	2.956(2)	175.7(19)	x,y+1,z
	N(8)-H(2N8)...O(4)	2.814(2)	147(2)	x,y+1,z
	N(7)-H(1N7)...O(5)	2.730(2)	150(2)	
	N(7)-H(1N7)...O(6)	3.076(2)	133.7(19)	
	O(8)-H(1O8)...O(9)	2.890(3)	173(3)	-x,-y+1,-z+2
	O(8)-H(1O8)...O(10)	2.914(2)	113(3)	-x,-y+1,-z+2
	O(17)-H(3O1)...O(1)	2.802(2)	158(3)	-x+1,-y,-z
	O(17)-H(3O1)...O(2)	3.311(2)	134(3)	-x+1,-y,-z
	O(17)-H(4O1)...O(2)	2.779(2)	167(3)	x,y+1,z
	O(16)-H(1O0)...O(17)	2.636(3)	163(3)	
pyr·smz·CH_3OH	N(5)-H(5A)...O(2)	3.137(8)	153.3	-x+2,y-1/2,-z+1/2
	N(5)-H(5B)...O(3)	3.116(9)	159.1	x+1,-y+1/2,z-1/2
	N(1)-H(2N1)...O(1)	2.877(7)	177(7)	
	N(3)-H(1N3)...O(3)	3.193(9)	133(4)	
	N(3)-H(2N3)...N(8)	3.020(8)	171(5)	
	N(6)-H(1N6)...N(2)	3.173(7)	172(5)	

$(pyr^+)_2(ox^{2-})\cdot 1.5H_2O$	N(6)-H(1N6)...N(3)	2.962(4)	172(4)	x+1/2,-y+3/2,z-1/2
	N(6)-H(2N6)...O(2)	3.010(4)	138(4)	x,-y+3/2,-z+1/2
	N(6)-H(2N6)...O(3)	2.923(4)	121(3)	
	N(1)-H(1N1)...O(4)	2.652(4)	165(4)	
	N(2)-H(1N2)...N(7)	3.045(4)	172(4)	x-1/2,-y+3/2,z+1/2
	N(2)-H(2N2)...O(1)	2.753(4)	156(5)	
	N(4)-H(2N4)...O(5)	2.839(4)	154(4)	x-1/2,y,-z+1
	N(5)-H(1N5)...O(2)	2.685(3)	165(4)	
	N(5)-H(1N5)...O(3)	3.159(4)	122(3)	
	N(8)-H(1N8)...O(4)	2.831(4)	171(4)	x+1/2,y,-z
	O(5)-H(1O5)...O(1)	2.718(4)	167(4)	
	O(5)-H(2O5)...O(3)	2.825(4)	167(5)	x,-y+3/2,-z+1/2
$(tmp^+)_2(ox^{2-})\cdot 2CH_3OH$	O(6)-H(6)...O(5)	2.7082(16)	172.5	
	C(13)-H(13B)...O(3)	3.577(2)	167.7	-x+1,-y+1,-z+2
	C(14)-H(14A)...O(4)	3.344(3)	162.3	x,-y+1,z+1/2
	N(3)-H(1N3)...N(1)	3.0387(19)	167.0(18)	-x,-y+1,-z+1
	N(3)-H(2N3)...O(5)	2.7900(18)	157.7(18)	x+1/2,-y+1/2,-z+1
	N(4)-H(1N4)...O(6)	2.9811(17)	153.2(17)	x-1/2,y+1/2,z
	N(4)-H(2N4)...O(6)	2.9034(17)	161.7(18)	-x+1/2,y+1/2,-z+3/2
	N(2)-H(1N2)...O(4)	2.6843(16)	140.6(19)	
	N(2)-H(1N2)...O(5)	2.9663(18)	139.5(19)	-x+1/2,-y+1/2,-z+1
$(tmp^+)_2(ox^{2-})\cdot 6.5H_2O$	N(2)-H(2A)...O(6)	3.102(5)	162.8	-x+2,-y+2,-z+1
	N(2)-H(2B)...O(5)	2.797(4)	153.2	-x,-y+1,-z+1
	N(4)-H(1N4)...N(3)	3.034(4)	167(4)	-x+2,-y+2,-z+1
	N(4)-H(2N4)...O(6)	2.939(5)	136(4)	
	N(1)-H(1N1)...O(4)	2.815(4)	132(3)	
	N(1)-H(1N1)...O(5)	2.968(5)	151(3)	-x,-y+1,-z+1
	O(6)-H(1O6)...O(8)	2.912(6)	150(7)	x+1,y+1,z
	O(6)-H(2O6)...O(5)	3.112(6)	141(6)	x+1,y+1,z
$(pyr^+)(az^-)$ form I	N(1)-H(1N1)...O(1)	2.639(2)	172(2)	
	N(2)-H(1N2)...O(2)	2.817(2)	176(2)	
	N(2)-H(2N2)...O(3)	3.028(2)	171(2)	-x+1,-y+1,-z+1
	N(4)-H(1N4)...O(3)	2.938(2)	139(2)	x+2,y+1,z
	N(4)-H(2N4)...N(3)	2.970(2)	174(2)	-x+3,-y+2,-z+1
	O(4)-H(1O4)...O(2)	2.502(3)	174(3)	x,-y+1,-z+1
$(pyr^+)(az^-)$ form II	N(4)-H(1N4)...O(3)	2.931(3)	139(3)	x+2,y,z-1
	N(4)-H(2N4)...N(3)	2.985(3)	170(3)	-x+3,-y+1,-z
	N(2)-H(1N2)...O(2)	2.821(3)	173(3)	
	N(2)-H(2N2)...O(3)	2.984(3)	167(2)	-x+1,-y+1,-z+1
	N(1)-H(1N1)...O(1)	2.659(3)	172(3)	
	O(4)-H(1O4)...O(2)	2.496(3)	177(4)	-x,-y+1,-z+1

tmp·az	N(3)-H(2N3)...O(4)	3.022(3)	170(3)	
	N(4)-H(1N4)...O(6)	2.860(3)	176(3)	x+1,y+2,z
	N(4)-H(2N4)...O(2)	3.101(3)	153(2)	-x+2,-y+2,-z+1
	N(4)-H(2N4)...O(3)	3.019(3)	116.8(19)	-x+2,-y+2,-z+1
	O(5)-H(1O5)...N(1)	2.573(3)	172(3)	
$(tmp^{+})(az^{-})$	O(4B^b)-H(4B^b)..O(2)	2.617(9)	172.5	-x+3/2,-y+1,z-1/2
	N(2)-H(1N2)...O(5)	3.080(4)	164(4)	x,-y+1/2,z-1/2
	N(2)-H(1N2)...O(6)	3.189(4)	128(3)	x,-y+1/2,z-1/2
	N(2)-H(2N2)...O(1)	2.836(4)	168(3)	
	N(1)-H(1N1)...O(2)	2.719(4)	171(3)	
	N(4)-H(1N4)...O(6)	2.945(4)	133(3)	x-1/2,y,-z+3/2
	N(4)-H(2N4)...O(1)	2.861(3)	167(3)	x-1/2,-y+1/2,-z+1
$(tmp^{+})_2(az^{2-})\cdot tmp\cdot 6H_2O$	C(12)-H(12A)...O(7)	3.462(3)	163.0	x,y+1,z
	N(1)-H(1N1)...O(16)	3.130(3)	162(2)	x,y+1,z
	N(1)-H(2N1)...O(9)	3.017(2)	159(2)	-x+1,-y+1,-z+2
	N(10)-H(3N0)...O(10)	3.024(3)	171(2)	
	N(10)-H(4N0)...O(16)	2.867(3)	162(2)	-x+1,-y,-z+2
	N(8)-H(1N8)...O(19)	3.039(3)	156(2)	x,y-1,z
	N(8)-H(2N8)...O(15)	2.995(3)	171(2)	x-1,y+1,z
	N(12)-H(3N2)...O(9)	2.894(2)	154.5(19)	x,y-1,z
	N(12)-H(4N2)...N(2)	2.996(2)	165(2)	-x+1,-y,-z+2
	N(3)-H(1N3)...O(8)	3.064(3)	161(2)	
	N(3)-H(2N3)...N(11)	3.399(3)	174(2)	-x+1,-y,-z+2
	N(5)-H(1N5)...O(17)	2.846(3)	161(2)	
	N(6)-H(1N6)...O(10)	2.922(3)	172(2)	x-1,y+1,z
	N(6)-H(2N6)...O(8)	2.901(2)	171(2)	
	O(17)-H(3O7)...N(4)	2.926(3)	171(4)	
	O(17)-H(4O7)...O(7)	2.693(3)	174(3)	
	O(16)-H(3O6)...O(8)	2.741(2)	172(3)	
	O(14)-H(3O4)...O(7)	2.697(2)	176(3)	x,y-1,z
	O(14)-H(4O4)...O(10)	2.658(3)	163(4)	
	O(16)-H(4O6)...O(9)	2.864(2)	169(3)	x-1,y+1,z
	O(19)-H(3O9)...O(5)	3.152(2)	129(3)	x-1,y+1,z
	O(19)-H(3O9)...O(6)	2.961(3)	151(4)	x-1,y+1,z
	O(15)-H(3O5)...O(14)	2.898(3)	176(5)	
	O(15)-H(4O5)...O(17)	3.184(3)	127(3)	x,y-1,z
	O(18)-H(3O8)...O(2)	3.339(3)	145(3)	
	O(18)-H(3O8)...O(3)	2.880(3)	148(3)	
	O(18)-H(4O8)...O(19)	2.780(3)	171(6)	
	O(19)-H(4O9)...O(18)	2.794(3)	163(5)	-x,-y+3,-z+1
	N(9)-H(1N9)...O(14)	2.802(2)	158(2)	
$pyr\cdot 0.5seb\cdot CH_3CN$	N(3)-H(1N3)...O(2)	2.904(3)	162(3)	
	N(3)-H(2N3)...N(5)	3.276(4)	150(2)	-x,-y+1,-z
	O(1)-H(1O1)...N(1)	2.715(2)	171(3)	
	N(4)-H(1N4)...N(5)	3.116(4)	145(2)	
	N(4)-H(2N4)...N(2)	3.019(3)	176(3)	-x,-y+1,-z

$(tmp^+)_2(seb^{2-})$ $\cdot 2CH_3OH \cdot 2H_2O$	N(1)-H(1N1)...O(4)	2.627(2)	173(2)	
	N(2)-H(1N2)...O(7)	3.150(3)	171.9(19)	-x,-y+1,-z
	N(2)-H(2N2)...O(5)	2.842(2)	170(2)	
	N(4)-H(1N4)...O(7)	2.842(2)	148(2)	x+1,y-1,z
	N(4)-H(2N4)...N(3)	2.967(2)	172.2(19)	-x+1,-y,-z
	O(7)-H(1O7)...O(6)	2.871(4)	167(3)	
	O(7)-H(2O7)...O(5)	2.771(2)	177(4)	
	O(6)-H(1O6)...O(2)	2.909(3)	152(5)	x-1,y,z
	O(6)-H(1O6)...O(3)	3.005(3)	134(5)	x-1,y,z
$tmp \cdot pim \cdot 0.5CH_3CN$	N(2)-H(2A)...O(1)	3.015(3)	166.1	
	N(2)-H(2B)...O(5)	2.964(3)	127.7	
	N(4)-H(1N4)...O(13)	3.053(3)	137(3)	x-1,y,z-1
	N(4)-H(2N4)...O(4)	2.894(3)	174(3)	x,y+1,z
	N(8)-H(1N8)...O(9)	2.939(3)	114(2)	x+1,y,z+1
	N(8)-H(1N8)...O(10)	3.065(3)	143(2)	x+1,y,z+1
	N(8)-H(2N8)...O(7)	2.917(3)	172(3)	x,y-1,z
	O(3)-H(1O3)...N(3)	2.675(3)	171(3)	x,y-1,z
	O(2)-H(1O2)...N(1)	2.585(3)	168(4)	
	O(6)-H(1O6)...N(5)	2.570(3)	176(5)	
	O(8)-H(1O8)...N(7)	2.679(3)	173(4)	x,y+1,z
	N(6)-H(1N6)...O(5)	3.003(3)	170(3)	
	N(6)-H(2N6)...O(1)	2.956(3)	128(2)	
$(tmp^+)(pim^-) \cdot stz$	N(7)-H(1N7)...N(4)	3.026(3)	174(2)	-x+1,-y+2,-z+1
	N(7)-H(2N7)...O(1)	2.959(3)	146(2)	-x+1,-y+2,-z+1
	N(5)-H(1N5)...O(4)	2.687(3)	174(3)	
	N(1)-H(1N1)...O(2)	3.212(3)	159(3)	x,y,z+1
	N(1)-H(2N1)...O(7)	3.287(4)	144(3)	x-1,y,z
	N(1)-H(2N1)...O(8)	3.139(4)	143(3)	x-1,y,z
	N(6)-H(1N6)...O(1)	3.261(3)	156(2)	
	N(6)-H(2N6)...O(3)	2.793(3)	173(3)	
	O(5)-H(1O5)...O(4)	2.614(3)	171(4)	x+1,-y+1,-z+1
	N(3)-H(1N3)...O(3)	2.649(3)	167(3)	
$(tmp^+)_2(seb^{2-}) \cdot 2stz$ $\cdot 2H_2O \cdot C_3H_6O$	N(1)-H(1N1)...O(7)	2.6818(18)	177(2)	
	N(2)-H(1N2)...O(6)	2.8817(19)	177(2)	
	N(2)-H(2N2)...O(5)	3.1101(19)	167(2)	
	N(2)-H(2N2)...N(6)	3.060(2)	111.5(17)	
	N(4)-H(1N4)...O(5)	2.7863(19)	142.2(16)	-x,-y+2,-z+1
	N(4)-H(2N4)...N(3)	3.027(2)	170(2)	-x,-y+2,-z+1
	O(9)-H(1O9)...S(1)	3.8190(15)	153(2)	-x+1,-y+2,-z+1
	O(9)-H(1O9)...O(4)	2.9457(19)	172(3)	-x+1,-y+2,-z+1
	O(9)-H(2O9)...O(7)	2.7130(19)	170(2)	
	N(5)-H(1N5)...O(9)	2.891(2)	172(3)	-x+1,-y+1,-z+1

Table S6. Solution crystallization screen of ternary cocrystals of tmp.

Coformer 1	Coformer 2	Solvent	Result
ox	stz	CH_3OH	crystallization of stz
		CH_3CN	small fibers
keto	stz	CH_3OH	thin fibers
		CH_3CN	oil
seb	stz	CH_3OH	fibers
		C_3H_6O	$(tmp^+)_2(seb^{2-})\cdot 2stz\cdot 2H_2O\cdot C_3H_6O$
az	stz	CH_3OH	crystallization of stz
		CH_3CN	tmp·az
pim	stz	CH_3OH	crystallization of stz
		CH_3CN	$(tmp^+)(pim^-)\cdot stz$
fum	stz	CH_3OH	$(tmp^+)_2(fum^{2-})$
		CH_3CN	powder
ox	smzo*	CH_3OH	$(tmp^+)_2(ox^{2-})\cdot 2CH_3OH$
		CH_3CN	powder
keto	smzo	CH_3OH	crystallization of smzo
		CH_3CN	powder
seb	smzo	CH_3OH	thin fibers
		CH_3CN	thin plates, not diffracting
az	smzo	CH_3OH	crystallization of smzo
		CH_3CN	tmp·az
pim	smzo	CH_3OH	crystallization of smzo
		CH_3CN	small needles
fum	smzo	CH_3OH	$(tmp^+)_2(fum^{2-})$
		CH_3CN	crystallization of smzo
ox	sgd**	CH_3OH	$(tmp^+)_2(ox^{2-})\cdot 2CH_3OH$
		CH_3CN	thin fibers
keto	sgd	CH_3OH	crystallization of sgd
		CH_3CN	$(tmp^+)(keto^-)\cdot 0.5H_2O$
seb	sgd	CH_3OH	thin fibers
		CH_3CN	crystallization of sgd
az	sgd	CH_3OH	crystallization of sgd
		CH_3CN	microcrystalline
pim	sgd	CH_3OH	thin fibers
		CH_3CN	soft fibers
fum	sgd	CH_3OH	powder
		CH_3CN	powder
ox	sxz***	CH_3OH	$(tmp^+)_2(ox^{2-})\cdot 2CH_3OH$
		CH_3CN	soft fibers
keto	sxz	CH_3OH	crystallization of sxz
		CH_3CN	$(tmp^+)(keto^-)\cdot 0.5H_2O$
seb	sxz	CH_3OH	crystallization of sxz

		CH_3CN	$(tmp^+)(seb^-)$ salt, heavily disordered, data not shown
az	sxz	CH_3OH	crystallization of sxz
		CH_3CN	powder, multicrystalline
pim	sxz	CH_3OH	crystallization of sxz
		CH_3CN	soft fibers
fum	sxz	CH_3OH	crystallization of sxz
		CH_3CN	powder
ox	sdx****	CH_3OH	$(tmp^+)_2(ox^{2-})\cdot 2CH_3OH$
		CH_3CN	thin fibers
keto	sdx	CH_3OH	crystallization of sdx
		CH_3CN	$(tmp^+)(keto^-)\cdot 0.5H_2O$
seb	sdx	CH_3OH	crystallization of sdx
		CH_3CN	$(tmp^+)(seb^-)$ salt, heavily disordered, data not shown
az	sdx	CH_3OH	crystallization of sdx
		CH_3CN	powder
pim	sdx	CH_3OH	crystallization of sdx
		CH_3CN	thin plates, not diffracting
fum	sdx	CH_3OH	crystallization of sdx

*smzo = sulfamethizole, **sgd = sulfaguanidine, ***sxz = sulfisoxazole, ****sdx = sulfadoxine

Table S7. Solution crystallization screen of ternary cocrystals of pyr .

Coformer 1	Coformer 2	Solvent	Result
sub	PASA*	CH_3OH	powder
		CH_3CN	$(pyr^+)(sub^-)$
seb	PASA	CH_3OH	multicrystalline
		CH_3CN	crystallization of PASA
ox	PASA	CH_3OH	multicrystalline
		CH_3CN	$(pyr^+)(ox^-)$
az	PASA	CH_3OH	powder
		CH_3CN	$(pyr^+)(az^-)$
fum	PASA	CH_3OH	known salt $(pyr^{+)}{}_2(fum^-)$ [1]
		CH_3CN	crystallization of PASA
pim	PASA	CH_3OH	crystallization of PASA
		CH_3CN	known salt $(pyr^+)(pim^-)$ [2]
keto	PASA	CH_3OH	oil
		CH_3CN	fibrous needles
ox	tmp	CH_3OH	$(tmp^+)_2(ox^{2-})\cdot 2CH_3OH$
		CH_3CN	thin fibers
		C_3H_6O	$(tmp^+)_2(ox^{2-})\cdot 6.5H_2O$
		$CH_3C(=O)OC_2H_5$	$(tmp^+)_2(ox^{2-})\cdot 6.5H_2O$
		CH_3OH/CH_3CN	$(pyr^+)_2(ox^{2-})\cdot 1.5H_2O$
keto	tmp	CH_3OH	powder
		CH_3CN	thin fibers
		C_3H_6O	thin fibers
		$CH_3C(=O)OC_2H_5$	thin fibers
		CH_3OH/CH_3CN	non-diffracting thin plates
sub	tmp	CH_3OH	crystallization of pyr
		CH_3CN	$pyr\cdot sub\cdot CH_3CN$
		C_3H_6O	powder
		$CH_3C(=O)OC_2H_5$	
		CH_3OH/CH_3CN	$(pyr^+)(sub^-)$
seb	tmp	CH_3OH	thin fibers
		CH_3CN	$pyr\cdot 0.5seb\cdot CH_3CN$
		C_3H_6O	powder
		$CH_3C(=O)OC_2H_5$	$(tmp^+)(seb^-)$ salt, heavily disordered, data not shown
		CH_3OH/CH_3CN	thin fibers and non-diffracting plates
az	tmp	CH_3OH	crystallization of pyr
		CH_3CN	$pyr\cdot CH_3CN$
		C_3H_6O	powder
		$CH_3C(=O)OC_2H_5$	$(tmp^+)(az^-)$
		CH_3OH/CH_3CN	oil

pim	tmp	CH_3OH	crystallization of pyr
		CH_3CN	powder
		C_3H_6O	powder
		$CH_3C(=O)OC_2H_5$	powder
		CH_3OH/CH_3CN	crystallization of pyr
pim	stz	CH_3OH	crystallization of stz
		CH_3CN	microcrystalline
seb	stz	CH_3OH	crystallization of stz
		CH_3CN	microcrystalline
sub	stz	CH_3OH	thin fibers
		CH_3CN	crystallization of stz
az	stz	CH_3OH	crystallization of stz
		CH_3CN	microcrystalline

*PASA = p-aminosalicylic acid.

[1] Sethuraman, V.; Stanley, N.; Muthiah, P. T.; Sheldrick, W. S.; Winter, M.; Luger, P.; Weber, M. *Cryst. Growth Des*. 2003, *3*, 823.

[2] Delori, A.; Galek, P.T.A.; Pidcock, E.; Jones, W. *Chem. Eur. J.* **2012**, *18*, 6835.

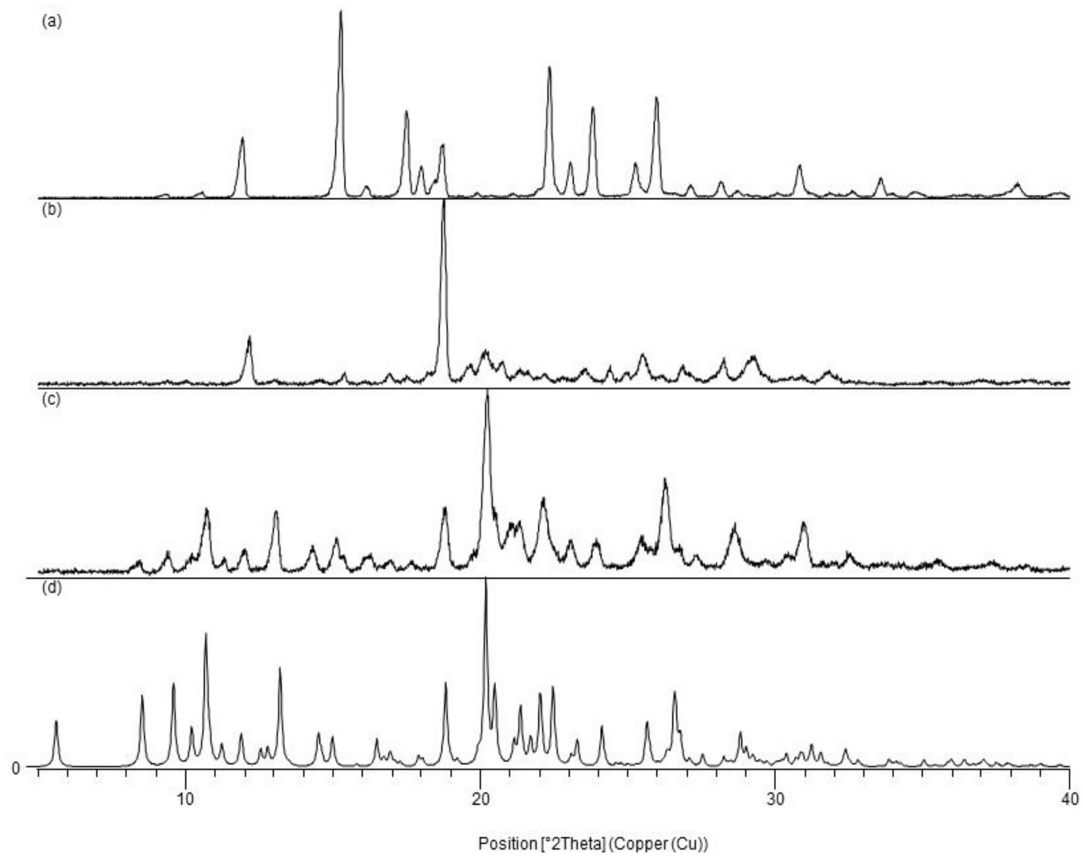

Figure S1. XRPD patterns of (a) tmp, (b) pyr, (c) a 1:1 mixture of tmp and pyr milled for 20 min at room temperature in the presence of traces of ethanol and (d) the theoretical XRPD pattern of the tmp·pyr·H_2O cocrystal calculated from the single crystal data.

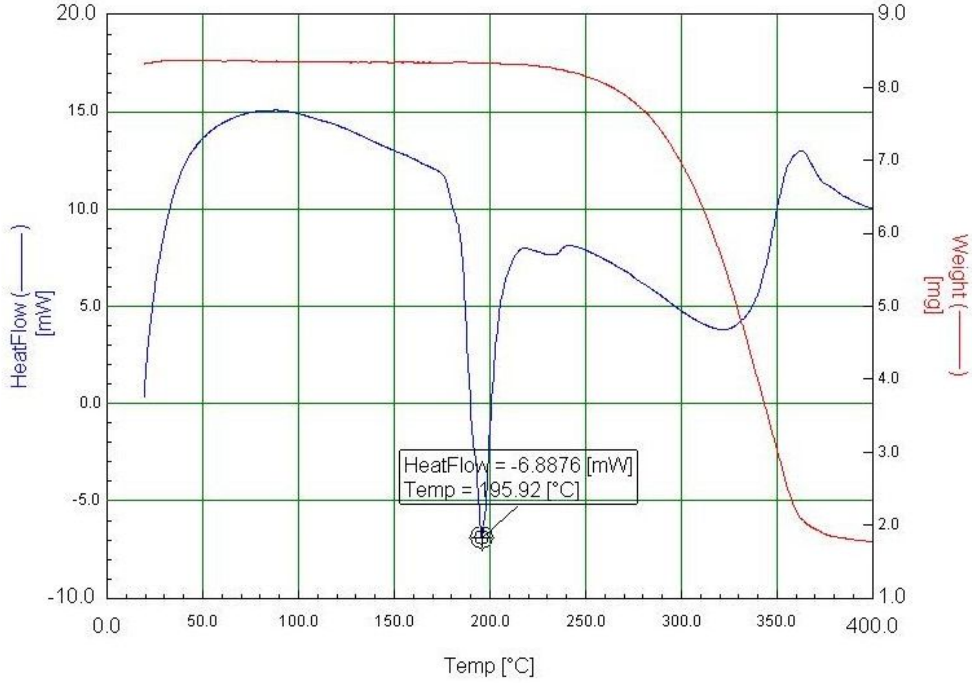

Figure S2. DSC plot of tmp·pyr·H_2O.

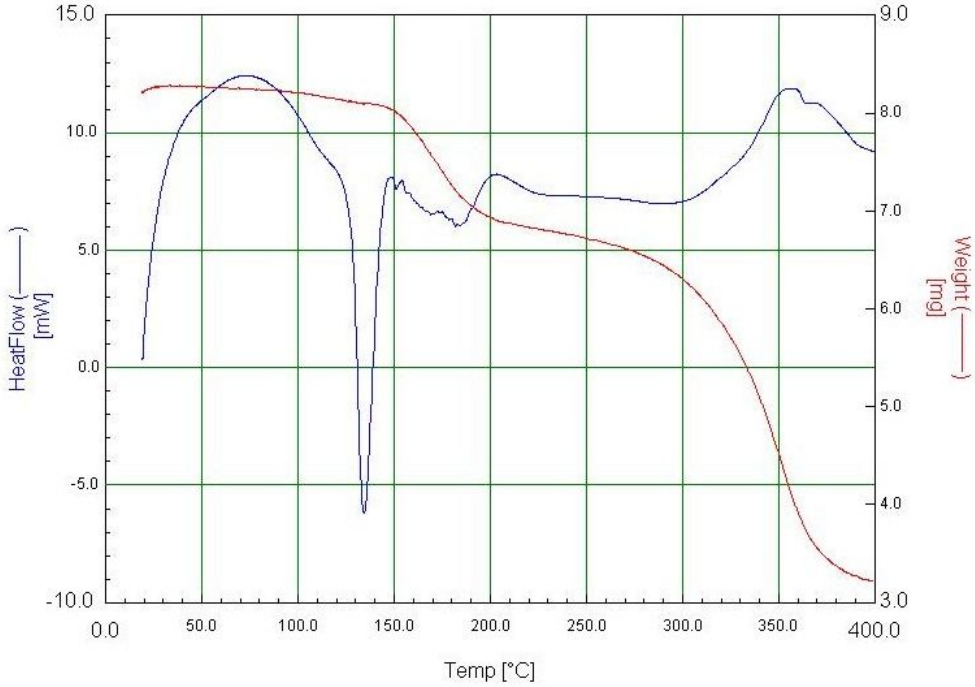

Figure S3. DSC plot of (tmp^+)($keto^-$)·0.5H_2O.

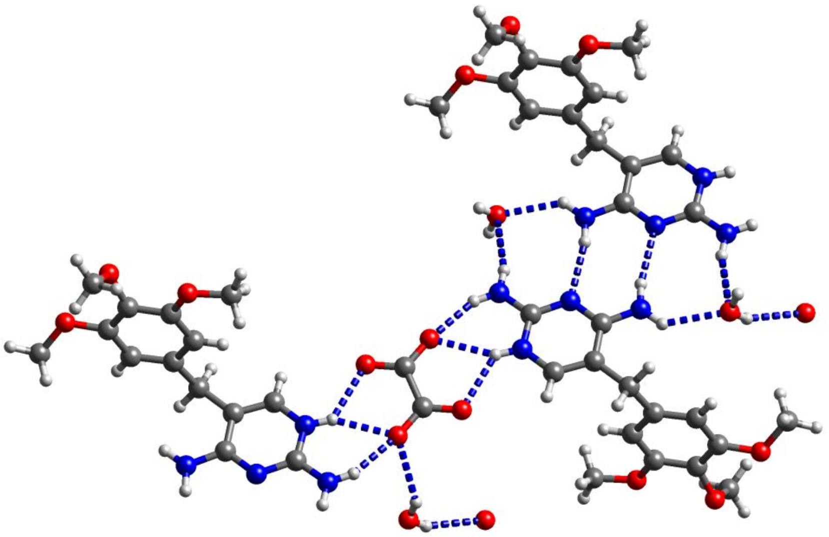

Figure S4. H bonding motif in $(tmp^{+})_2(ox^{2-})\cdot 6.5H_2O$. Only one component of the disordered trimethoxyphenyl ring is shown.

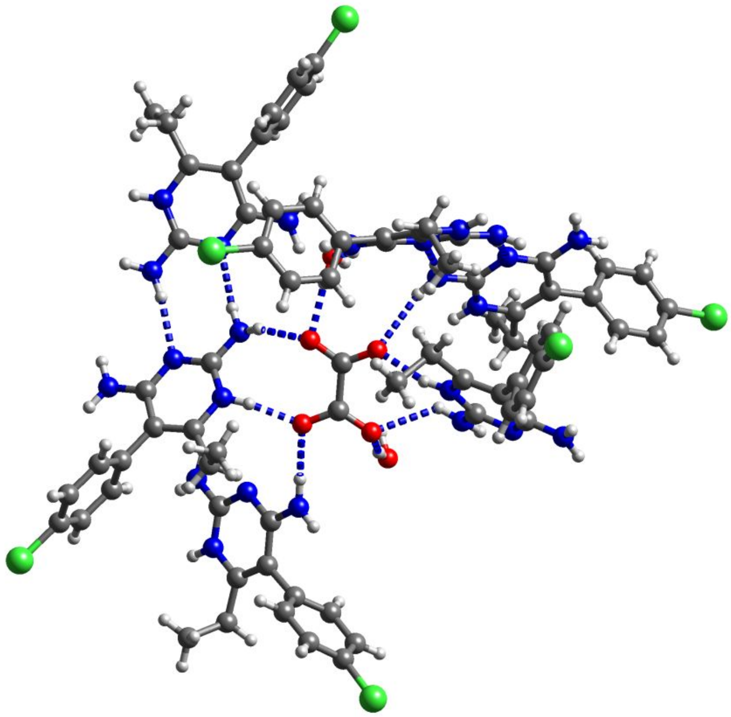

Figure S5. H bonding motif in $(pyr^{+})_2(ox^{2-})\cdot 1.5H_2O$. Only one component of the disordered ethyl group is shown.

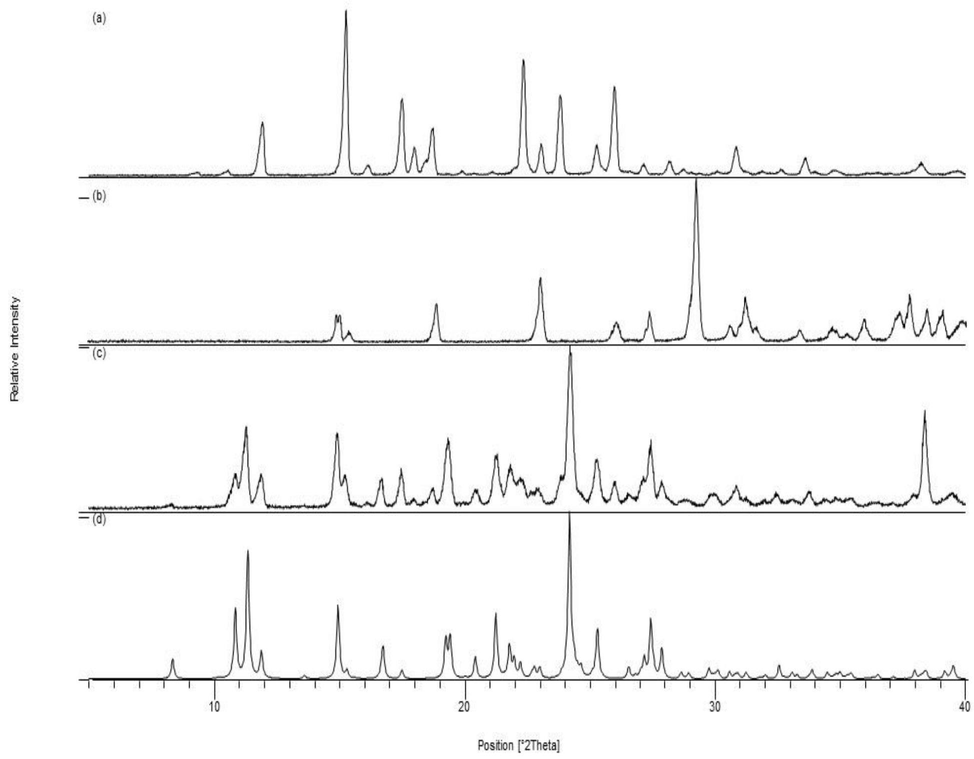

Figure S6. XRPD patterns of (a) tmp, (b) ox, (c) a 2:1 mixture of tmp and ox milled for 20 min at room temperature in the presence of traces of methanol and (d) the theoretical XRPD pattern of $(tmp^{+})_2(ox^{2-})\cdot 2CH_3OH$ calculated from the single crystal data.

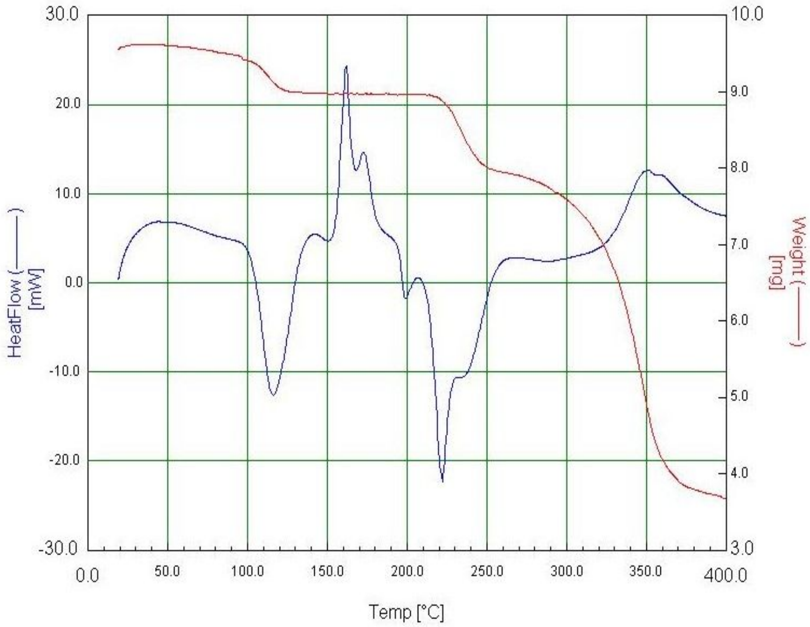

Figure S7. DSC plot of $(tmp^{+})_2(ox^{2-})\cdot 2CH_3OH$.

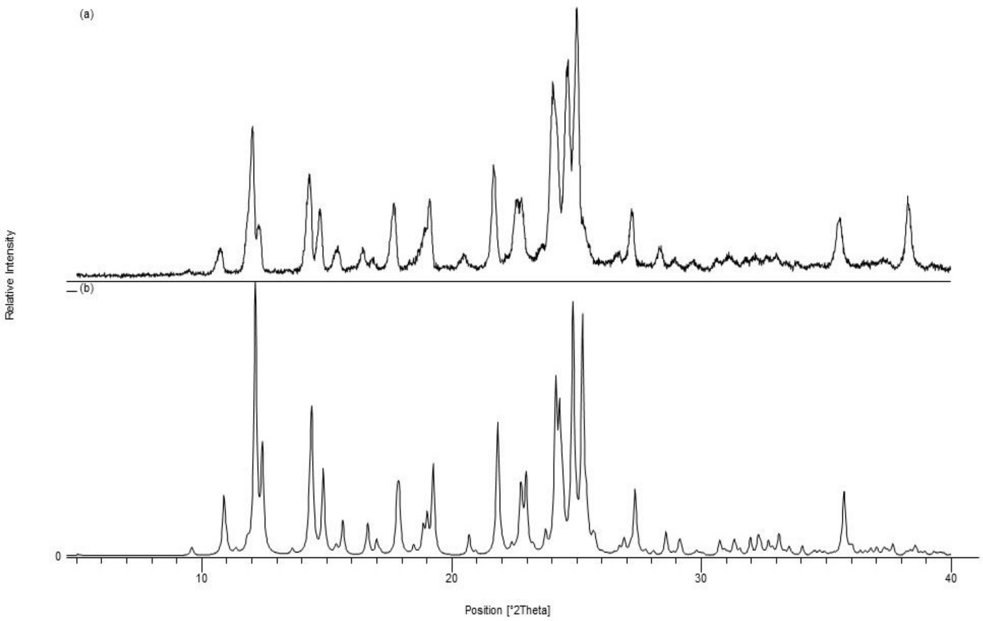

Figure S8. (a) XRPD pattern of the tmp·az crystals isolated from solution and (b) the theoretical XRPD pattern of tmp·az calculated from the single crystal data.

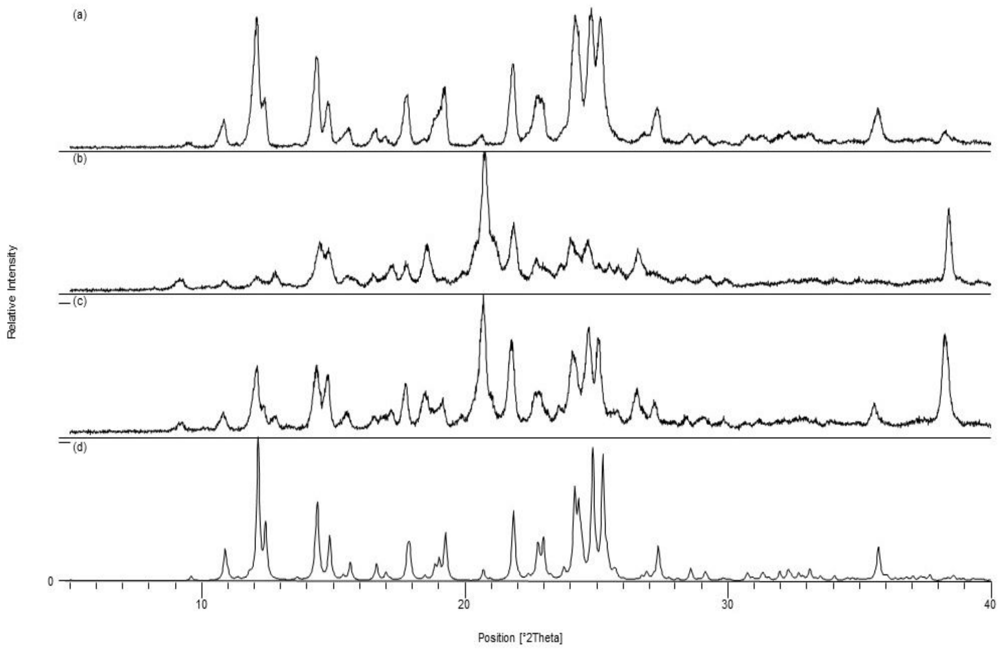

Figure S9. XRPD patterns of a 1:1 mixture of tmp and az after milling for 20 min at room temperature in the presence of (a) ethanol, (b) acetonitrile and (c) ethyl acetate. (d) The theoretical XRPD pattern of tmp·az calculated from the single crystal data.

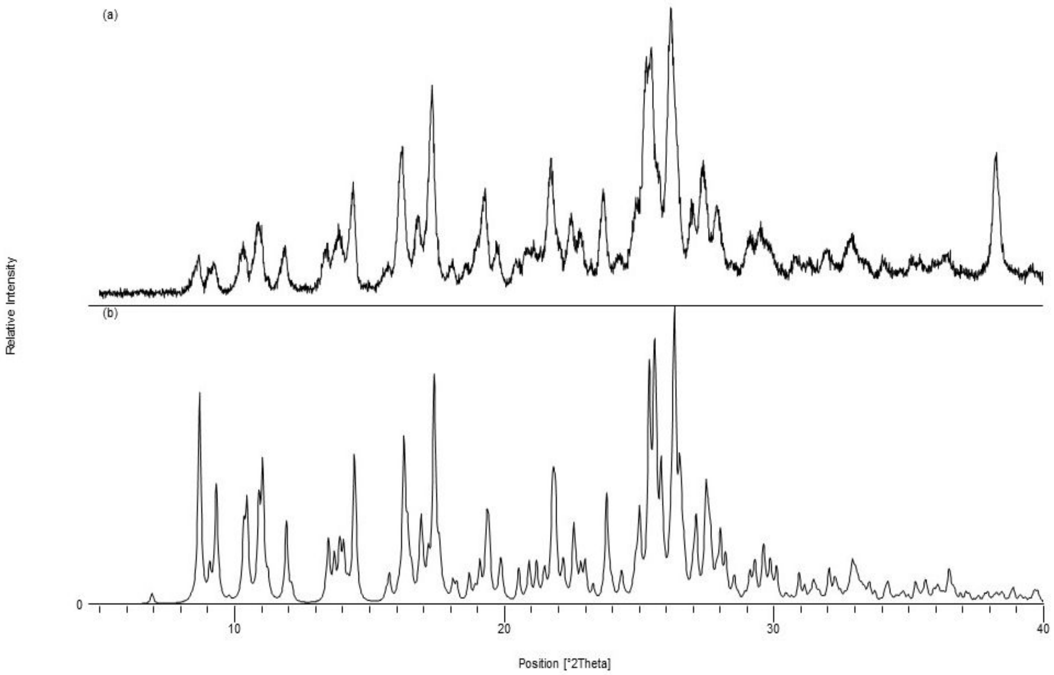

Figure S10. (a) XRPD pattern of a 3:1 mixture of tmp and az after milling for 20 min at room temperature in the presence of traces of water. (b) The theoretical XRPD pattern of $(tmp^{+})_2(az^{2-})\cdot tmp\cdot 6H_2O$ calculated from the single crystal data.

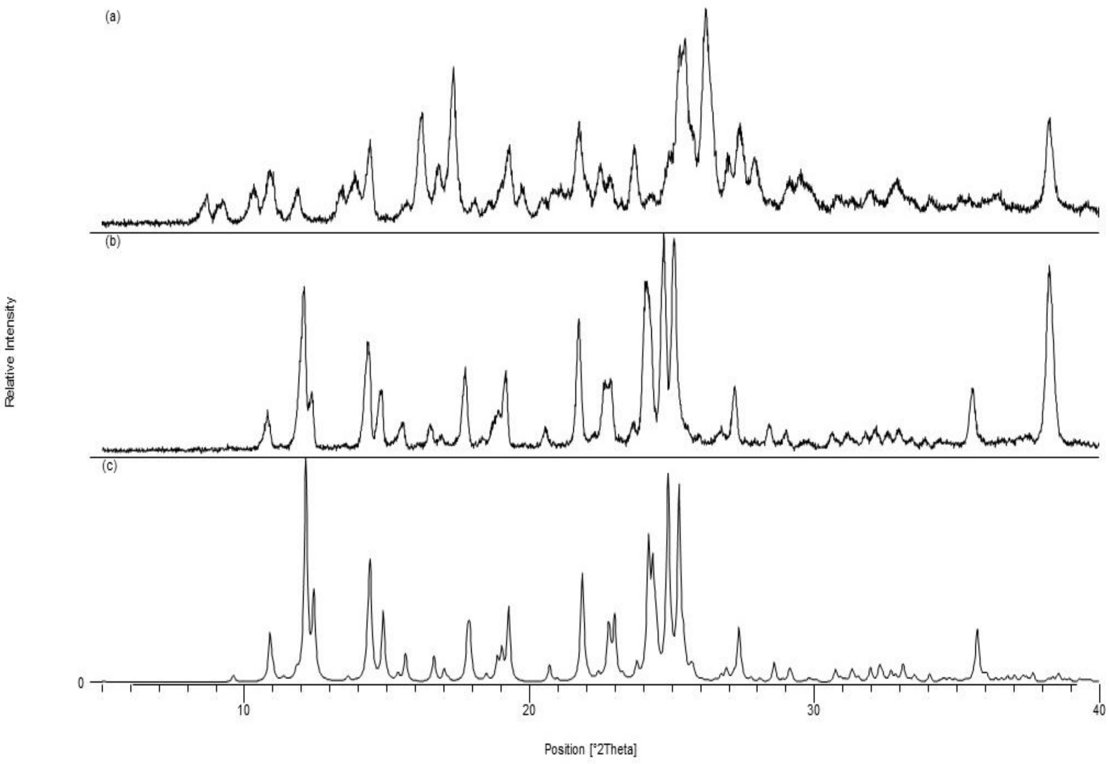

Figure S11. XRPD pattern of (a) $(tmp^{+})_2(az^{2-})$·tmp·$6H_2O$ and (b) after slurrying in methanol for 48 h at r.t. (c) The theoretical XRPD pattern of tmp·az calculated from the single crystal data.

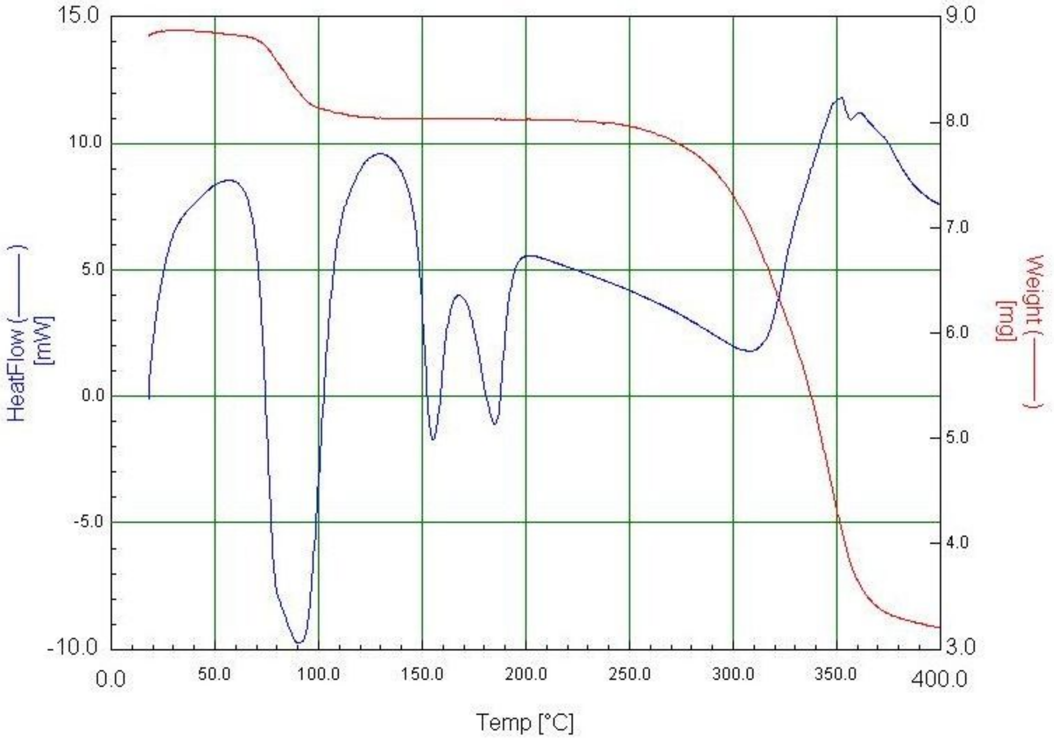

(a)

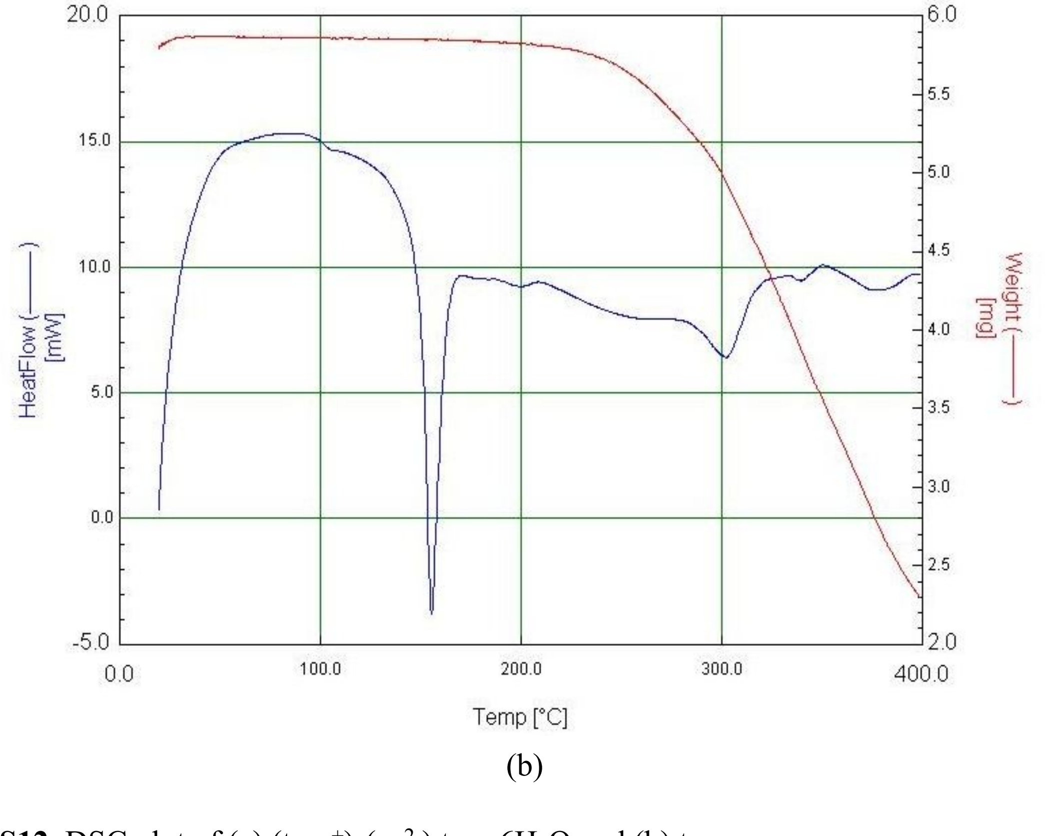

(b)

Figure S12. DSC plot of (a) $(tmp^{+})_2(az^{2-})$·tmp·$6H_2O$ and (b) tmp·az.

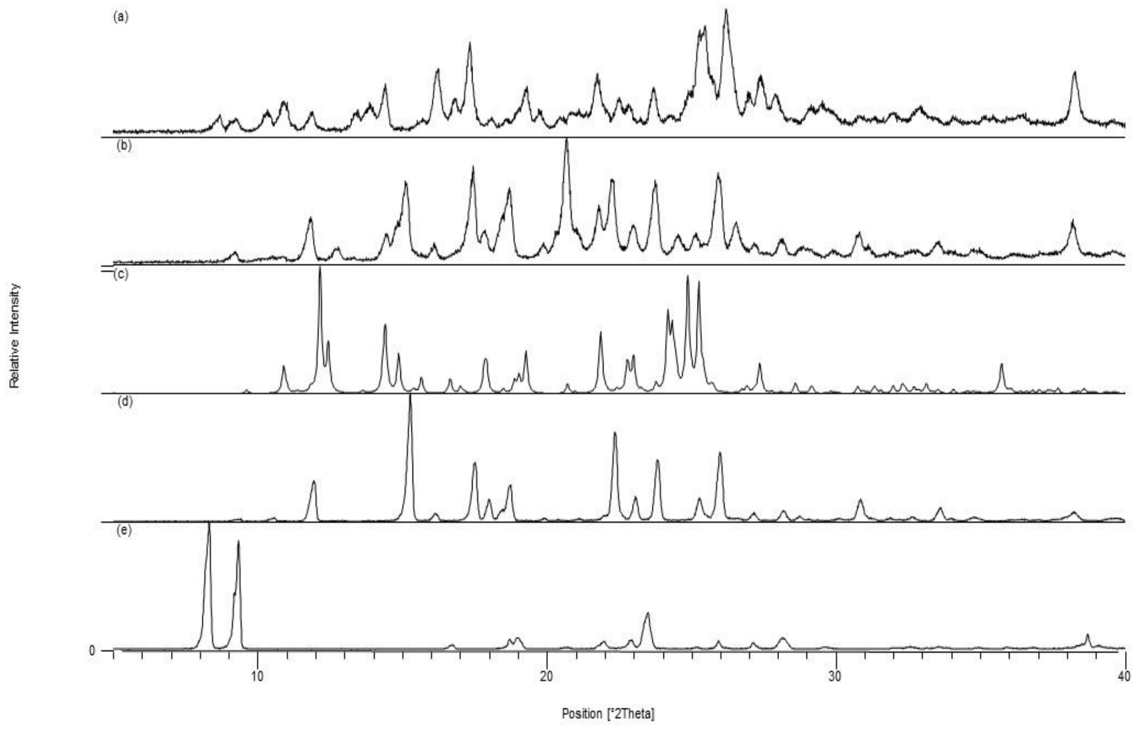

Figure S13. XRPD pattern of (a) $(tmp^+)_2(az^{2-})\cdot tmp\cdot 6H_2O$ and (b) after heating to 90 °C under vacuum for 6 h. (c) The theoretical XRPD pattern of tmp·az calculated from the single crystal data. (d) XRPD pattern of tmp, (e) XRPD pattern of az.

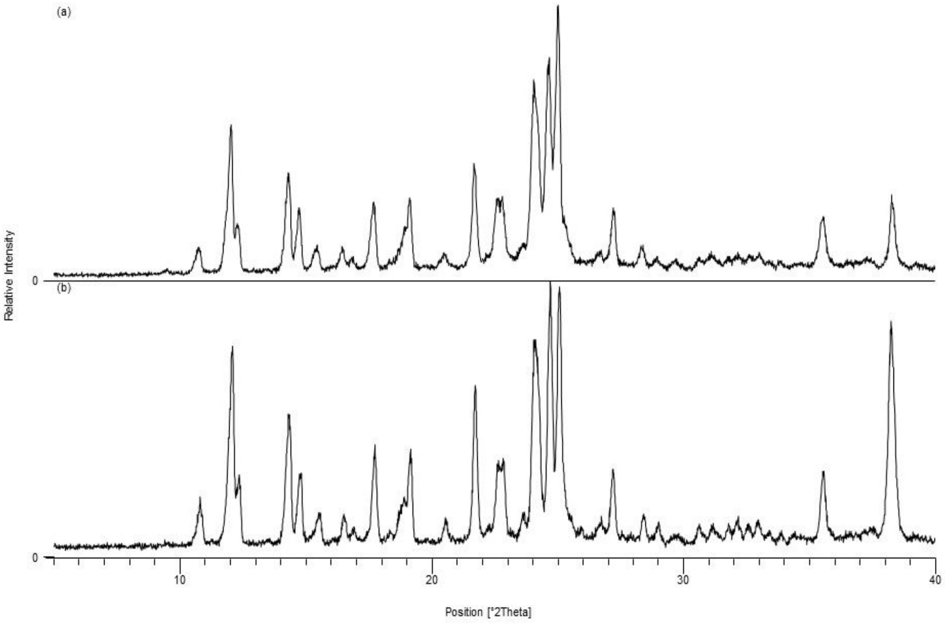

Figure S14. XRPD pattern of (a) tmp·az and (b) after slurrying in methanol for 48 h at r.t.

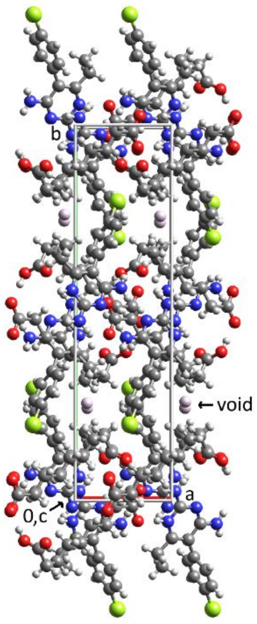

Figure S15. Unit cell of $(pyr^{+})(az^{-})$ with voids indicated as purple spheres.

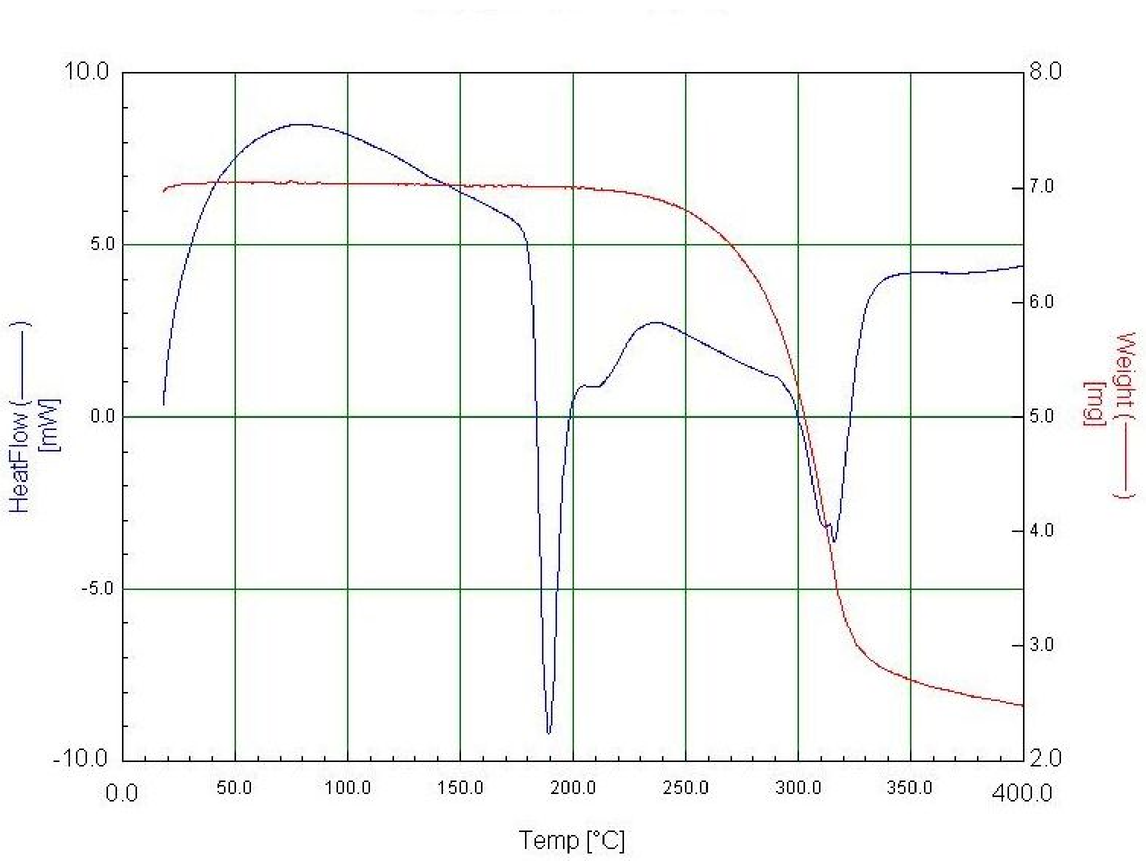

Figure S16. DSC plot of pyr·smz·CH_3OH.

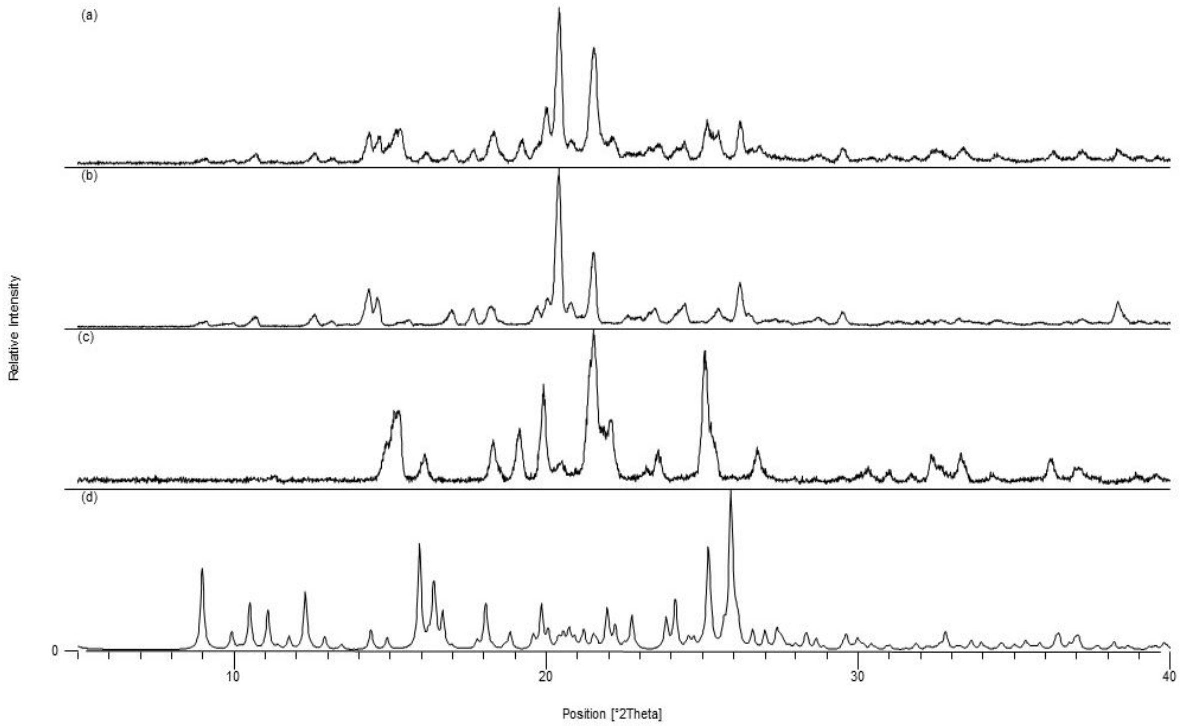

Figure 17. XRPD of (a) a 1:1:1 mixture of tmp, seb and stz milled for 20 min at room temperature in the presence of traces of acetone, (b) a 1:1 mixture of tmp and seb milled for 20 minutes in the presence of traces of methanol, (c) stz milled with traces of ethanol and (d) the theoretical XRPD pattern of $(tmp^{+})_2(seb^{2-})\cdot stz\cdot 2H_2O\cdot C_3H_6O$ calculated from the single crystal data.

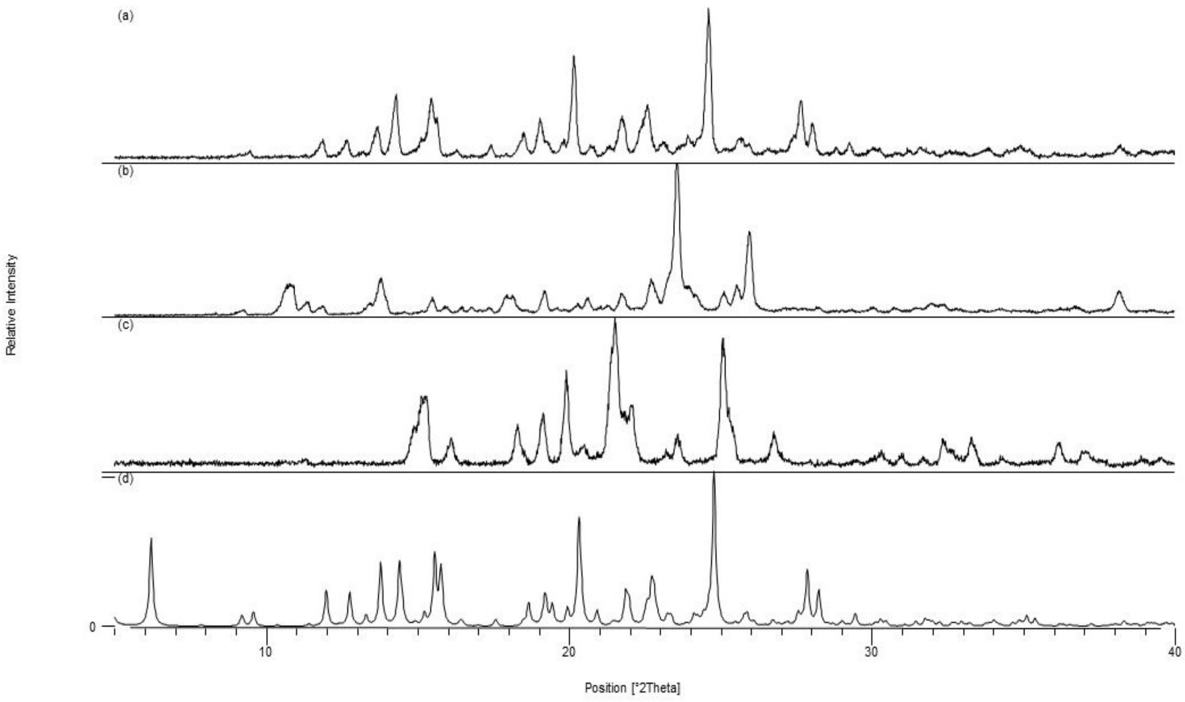

Figure 18. XRPD pattern of (a) a 1:1:1 mixture of tmp, pim and stz milled for 20 min at room temperature in the presence of traces of acetonitrile, (b) a 1:1 mixture of tmp and pim milled for 20 minutes in the presence of traces of acetonitrile, (c) stz milled with traces of ethanol and (d) the theoretical XRPD pattern of (tmp^+)(pim^-)·stz calculated from the single crystal data.

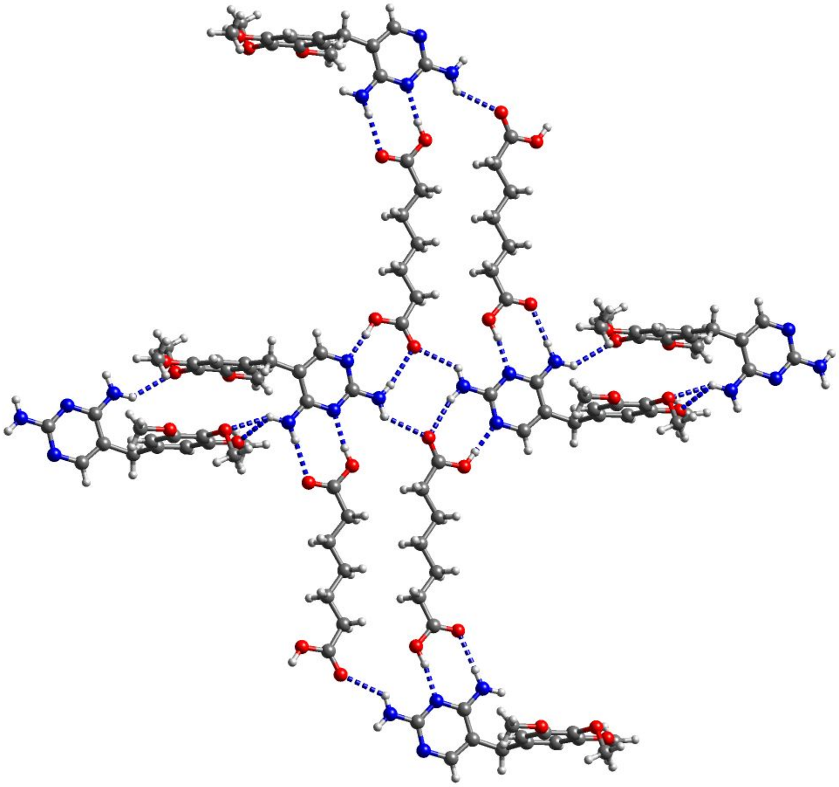

Figure S19. H bonding motif in tmp·pim·0.5CH_3CN. The solvent molecule of crystallization is not shown.
